# H7N9 virulent mutants detected in chickens in China pose an increased threat to humans

**DOI:** 10.1038/cr.2017.129

**Published:** 2017-10-24

**Authors:** Jianzhong Shi, Guohua Deng, Huihui Kong, Chunyang Gu, Shujie Ma, Xin Yin, Xianying Zeng, Pengfei Cui, Yan Chen, Huanliang Yang, Xiaopeng Wan, Xiurong Wang, Liling Liu, Pucheng Chen, Yongping Jiang, Jinxiong Liu, Yuntao Guan, Yasuo Suzuki, Mei Li, Zhiyuan Qu, Lizheng Guan, Jinkai Zang, Wenli Gu, Shuyu Han, Yangming Song, Yuzhen Hu, Zeng Wang, Linlin Gu, Wenyu Yang, Libin Liang, Hongmei Bao, Guobin Tian, Yanbing Li, Chuanling Qiao, Li Jiang, Chengjun Li, Zhigao Bu, Hualan Chen

**Affiliations:** 1 State Key Laboratory of Veterinary Biotechnology, Harbin Veterinary Research Institute, Chinese Academy of Agricultural Sciences, Harbin 150001, China;; 2 College of Life and Health Sciences, Chubu University, Aichi 487-8501, Japan

**Keywords:** influenza virus, H7N9, highly pathogenic, pandemic potential, transmission

## Abstract

Certain low pathogenic avian influenza viruses can mutate to highly pathogenic viruses when they circulate in domestic poultry, at which point they can cause devastating poultry diseases and severe economic damage. The H7N9 influenza viruses that emerged in 2013 in China had caused severe human infections and deaths. However, these viruses were nonlethal in poultry. It is unknown whether the H7N9 viruses can acquire additional mutations during their circulation in nature and become lethal to poultry and more dangerous for humans. Here, we evaluated the evolution of H7N9 viruses isolated from avian species between 2013 and 2017 in China and found 23 different genotypes, 7 of which were detected only in ducks and were genetically distinct from the other 16 genotypes that evolved from the 2013 H7N9 viruses. Importantly, some H7N9 viruses obtained an insertion of four amino acids in their hemagglutinin (HA) cleavage site and were lethal in chickens. The index strain was not lethal in mice or ferrets, but readily obtained the 627K or 701N mutation in its PB2 segment upon replication in ferrets, causing it to become highly lethal in mice and ferrets and to be transmitted efficiently in ferrets by respiratory droplet. H7N9 viruses bearing the HA insertion and PB2 627K mutation have been detected in humans in China. Our study indicates that the new H7N9 mutants are lethal to chickens and pose an increased threat to human health, and thus highlights the need to control and eradicate the H7N9 viruses to prevent a possible pandemic.

## Introduction

Influenza viruses are negative-sense RNA viruses, whose genome comprises eight gene segments: basic polymerase 2 (PB2), basic polymerase 1 (PB1), acidic polymerase (PA), hemagglutinin (HA), nucleoprotein (NP), neuraminidase (NA), matrix (M), and nonstructural protein (NS). Each gene segment encodes one or two proteins. Influenza A viruses are categorized into different subtypes on the basis of antigenic differences in their two surface glycoproteins: HA and NA. Currently, 16 different HA and 9 different NA subtypes of influenza viruses have been detected in avian species, but only three subtypes, namely H1N1, H2N2, and H3N2, have caused influenza pandemics in humans^1,2^.

In the last two decades, avian influenza viruses of the H5N1 and H7N9 subtypes have continued to present challenges to the poultry industry and human health. The H5N1 highly pathogenic avian influenza viruses have caused infections and disease outbreaks among poultry and wild birds in over 60 countries around the world^3,4^, and have sporadically jumped to humans and caused severe disease and deaths^5^. Several studies have indicated that the H5N1 influenza viruses will become transmissible in mammals if they acquire more mutations in their HA protein that allow them to recognize human-type receptors and the mutation of lysine (K) at position 627 of their PB2^6,7,8^, or if they reassort with human influenza viruses^9^.

H7N9 subtype viruses have caused severe human infections and deaths every year in China since they emerged in 2013^10^. Epidemiology studies have shown that humans become infected mainly through exposure to virus-infected poultry or a contaminated environment^11,12,13,14,15^. Biologic studies on H7N9 viruses have revealed several important characters: (i) the viruses can replicate efficiently in chickens but do not cause disease in any avian species^16^; (ii) most of the viruses can bind human-type receptors^16,17,18,19^, mainly because they bear valine (V) at position 186 and leucine (L) at position 226 in their HA protein^18^ (H3 numbering used throughout), which is an important determinant for avian influenza virus to infect humans; and (iii) when the viruses replicate in humans, they can easily obtain the glutamic acid (E) to K mutation at position 627 (E627K) or aspartic acid (D) to asparagine (N) mutation at position 701 (D701N) in their PB2^10,11,16,20,21^. These two mutations are known to increase the virulence and transmissibility of avian influenza viruses in mammals^22,23,24,25,26,27^. Indeed, the human H7N9 viruses are transmissible in ferrets, although their transmissibility varies among different strains^16,17,28,29,30^. The ability to bind to human-type receptors and transmit in mammals is an important indicator of the pandemic potential of the H7N9 viruses. However, because the H7N9 viruses showed low pathogenicity in poultry, strategies to eradicate them in poultry have not been successful in China, with the exception of the temporary closure of live poultry markets in cities where human cases were detected.

The low pathogenic H5 and H7 avian influenza viruses are predisposed to acquire more mutations when they circulate in gallinaceous poultry and subsequently become highly pathogenic for chickens and turkeys, as occurred of the H5N2 outbreak in the United States in 1983^31^, the H5N2 outbreak in Mexico in 1995^32^, and the H7N1 outbreak in Italy in 1999^33^. It is unknown whether the H7N9 viruses can acquire additional mutations during their circulation in nature and become lethal to poultry. Therefore, close monitoring and evaluation of the H7N9 viruses have important implications for both animal and human public health. Here, we characterized H7N9 viruses isolated from avian species in China between 2013 and 2017.

## Results

### Surveillance and genetic analysis of H7N9 avian influenza viruses

To monitor the evolution of the H7N9 viruses in poultry, we collected 112 593 samples from poultry markets, farms, wild bird habitats, and slaughterhouses in 24 provinces from July 2013 to January 2017, and inoculated the samples individually into 10-day-old embryonated chicken eggs for virus isolation. In total, 3 664 influenza viruses were detected from these samples, of which 293 strains of H7N9 viruses were isolated across 17 provinces (Supplementary information, Table S1). Six viruses were isolated from samples collected from chicken farms in Fujian, Guangdong, Jiangsu, and Zhejiang provinces; all of the other viruses were isolated from samples collected from live poultry markets (Supplementary information, Table S1).

To detect the key mutations that may significantly affect the biologic properties of the viruses, portions of the HA and PB2 genes of the 293 viruses were sequenced. We found that 280 and 266 of the 293 avian viruses bear 186V and 226L mutations, respectively, in their HA, but none of them bear the 627K or 701N mutations in their PB2 (Table 1). Importantly, 12 extra nucleotides (-aaacggactgcg-) encoding four amino acids (-KRTA-) in the cleavage site of HA were detected in seven viruses isolated from chickens in Guangdong province in 2017 (Table 1).

**Figure 1:**
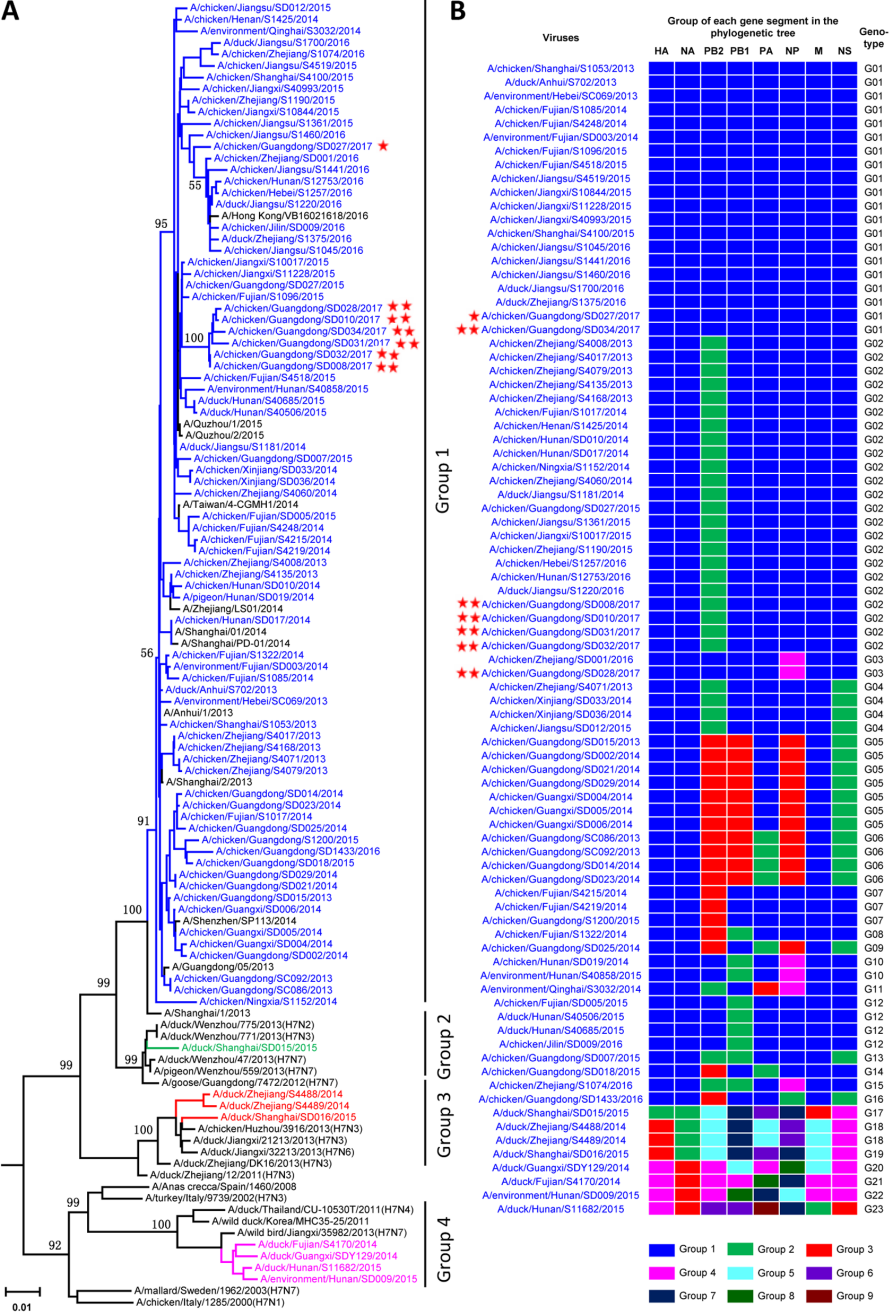
Genetic relationships among the HA genes and genotype evolution of H7N9 influenza viruses. **(A)** Phylogenetic tree of HA. The tree was rooted to A/chicken/Rostock/45/1934(H7N1) virus and based on an alignment of HA0 (nucleotides 29-1 723 of the viruses with stars and nucleotides 29-1 711 of the others). Sequences of viruses with names in black were downloaded from available databases; viruses with names in colors were sequenced in this study. The scale bar indicates the number of nucleotide substitutions per site. **(B)** Genotypes of the H7N9 viruses. The eight gene segments are indicated at the top of each bar. The colors of the bars represent the groups in the trees of Figure 1A and Supplementary information, Figure S1. Viruses labeled with red star(s) contain the four-amino acid (-KRTA-) insertion in their HA genes.

To investigate the genetic relationships among these viruses, we sequenced 83 representative viruses from different sampling times, places, and species. The HA genes of these viruses shared 88%-100% identity at the nucleotide level and formed four phylogenetic groups (Figure 1A). The HA genes of the group 1 viruses shared over 97% identity and clustered with the HA genes of the 2013/H7N9 viruses, whereas the HA genes of the other three groups belonged to viruses that emerged in China during this time period and were detected only in ducks.

The NA genes of these viruses shared 90%-100% identity at the nucleotide level and formed three phylogenetic groups (Supplementary information, Figure S1A). The six internal genes of these H7N9 viruses showed distinct diversity, with the PB2, PB1, PA, NP, M, and NS genes of the 83 viruses sharing 84.8%-100%, 88.6%-100%, 86.8%-100%, 86.4%-100%, 88.5%-100%, and 87.8%-100% identity, respectively, at the nucleotide level. The PB1 and NP genes each formed eight groups in the phylogenetic trees (Supplementary information, Figure S1C and S1E), and the PB2, PA, M, and NS genes each formed six, nine, five, and four groups, respectively, in their phylogenetic trees (Supplementary information, Figure S1B, S1D, S1F, and S1G).

Given this genomic diversity, we divided these H7N9 viruses into 23 different genotypes (Figure 1B). The predominant viruses were categorized as genotype 1 and 2 viruses because they were detected in eight and nine provinces, respectively; the viruses in the other genotypes were only detected in one to three provinces (Figure 1B; Supplementary information, Figure S3). The seven viruses bearing the four amino acid insertion in their HA belonged to genotypes 1, 2, and 3 (Figure 1B). Of note, the time-scaled phylogenetic analysis suggested that the HA mutants may have arisen from two different H7N9 viruses (Supplementary information, Figure S2), one of which subsequently reassorted with others to form two more genotypes (Figure 1, viruses labeled with two stars).

### Replication and virulence of H7N9 HA mutant A/chicken/Guangdong/SD008/2017 (CK/SD008) in chickens

**Figure 2:**
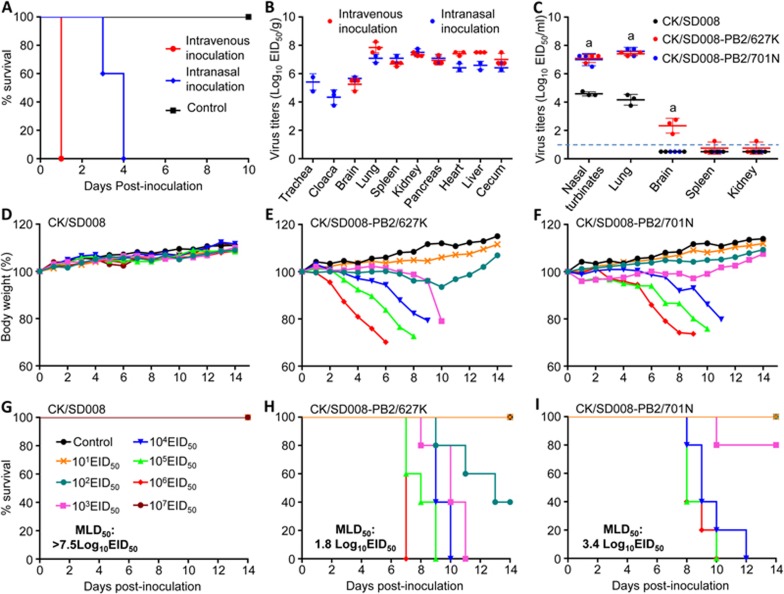
Replication and virulence of H7N9 viruses in chickens and mice. Death **(A)** and viral titers in organs **(B)** of chickens inoculated with CK/SD008. Viral titers in mice inoculated with CK/SD008, CK/SD008-PB2/627K, or CK/SD008-PB2/701N **(C)**. Body weight change **(D**-**F)** and death **(G**-**I)** of mice inoculated with CK/SD008 **(D**, **G)**, CK/SD008-PB2/627K **(E**, **H)**, or CK/SD008-PB2/701N **(F**, **I)**. The values or viral titers in mice were statistically analyzed by using a one-tailed paired *t*-test. ^a^*P* < 0.01 compared with the corresponding value for the CK/SD008-inoculated group. The dashed lines indicate the lower limit of detection.

The minimum motif in the HA cleavage site associated with high pathogenicity of H5 and H7 influenza viruses in chickens is -BXBR- (B = basic amino acids arginine or lysine, X = any amino acid, R = arginine)^34^ or -RXXR-^35^. The motif in the new H7N9 viruses is “-KRTAR-”, which meets the criterion of “-RXXR-” for high pathogenicity. Since not all H5 or H7 viruses that bear these motifs are highly pathogenic to chickens^36,37^, and the H7N9 mutants were all isolated from apparently healthy chickens in live poultry markets, we investigated their virulence by testing the intravenous pathogenicity index (IVPI) of the index strain A/chicken/Guangdong/SD008/2017 (CK/SD008)^38^. Groups of 10 6-week-old specific pathogen-free (SPF) chickens were inoculated intravenously with 0.1 ml of a 1:10 dilution of bacteria-free allantoic fluid containing 10^7.7^ 50% egg infectious dose (EID_50_) of the virus. All of the chickens inoculated with the virus died within 24 h post-inoculation (p.i.) (Figure 2A), yielding an IVPI value of 3 (0 = least pathogenic; 3 = most pathogenic). We collected brains, lungs, spleens, kidneys, pancreases, hearts, livers, and cecum from three chickens that were inoculated with CK/SD008 and determined viral titers in eggs. The virus was detected in all of these organs with mean titers ranging from 5.3 to 7.9 log_10_EID_50_/g (Figure 2B). We also inoculated 13 SPF chickens intranasally (i.n.) with 10^6^ EID_50_ of CK/SD008. Three chickens were killed on day 3 p.i. for virus titration and the other 10 were observed for signs of disease and death. The virus replicated systemically in chickens after i.n. inoculation and was detected in both pharyngeal and cloacal swabs (Figure 2B); the observed chickens died within 4 days of infection (Figure 2A). Our results indicate that an H7N9 virus isolated in 2017 that descended from the H7N9 viruses that emerged in China in 2013 has mutated into a highly pathogenic strain for chickens.

### Replication and virulence of H7N9 virus CK/SD008 in mice

The multiple basic amino acid motif in the HA cleavage site of H5N1 influenza viruses is a prerequisite for lethality in mammals^25,39^, but not all H5N1 viruses that have this motif are lethal in these animals^40,41^. To investigate whether the four amino-acid insertion in HA also increases H7N9 virus virulence in mammals, we tested the replication and virulence of virus with this HA insertion in mice and ferrets.

Three 6-week-old BALB/c mice were inoculated i.n. with 10^6.0^ EID_50_ of CK/SD008 and then killed 3 days p.i. Their nasal turbinates, lungs, spleens, kidneys, and brains were collected for virus titration. The 50% mouse lethal dose (MLD_50_) of the virus was determined by inoculating groups of five mice i.n. with 10^1.0^-10^7.0^ EID_50_ of the virus, and daily monitoring of body weight, disease signs, and death for 2 weeks. Virus replication was detected in the nasal turbinates and lungs of mice but not in their spleens, kidneys, or brains (Figure 2C). No disease signs or deaths were observed among mice inoculated with any dose of the virus, and all mice gained weight during the observation period (Figure 2D). Therefore, the MLD_50_ of CK/SD008 is > 7.5log_10_EID_50_ (Figure 2G).

### Replication and transmission of the CK/SD008 virus in ferrets

**Figure 3:**
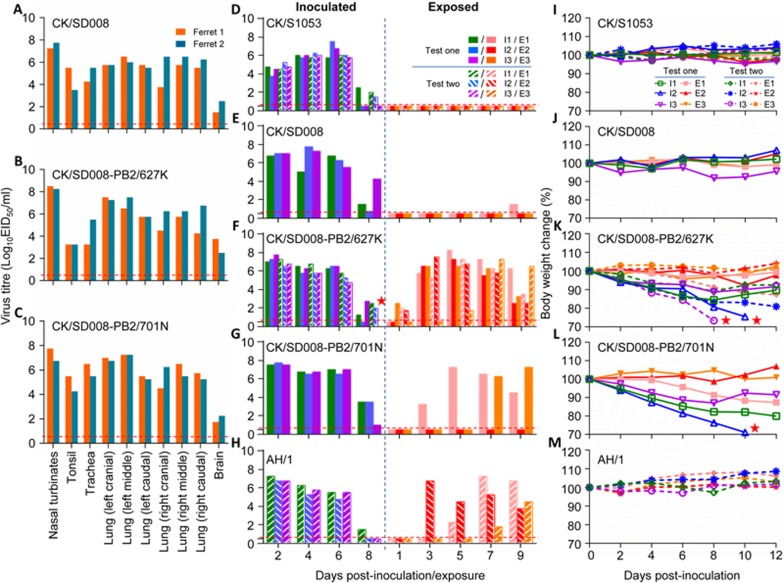
Replication and respiratory droplet transmission of H7N9 viruses in ferrets. Virus replication: **(A)** CK/SD008; **(B)** CK/SD008-PB2/627K; **(C)** CK/SD008-PB2/701N. Virus respiratory droplet transmission: **(D)** CK/S1053; **(E)** CK/SD008; **(F)** CK/SD008-PB2/627K; **(G)** CK/SD008-PB2/701N; **(H)** AH/1. Body weight change: **(I)** CK/S1053; **(J)** CK/SD008; **(K)** CK/SD008-PB2/627K; **(L)** CK/SD008-PB2/701N; **(M)** AH/1. Each bar **(A-H)** represents the virus titers from an individual animal, and the dashed red lines indicate the lower limit of detection. The red stars on **F**, **K**, and **L** indicate the day the animal died.

To evaluate viral replication in ferrets, we inoculated two ferrets i.n. with 10^6.0^ EID_50_ of CK/SD008. Nasal turbinates, tonsils, trachea, lungs, spleen, kidneys, and brain were collected on day 4 p.i. for virus titration in eggs. Unlike the 2013 H7N9 avian viruses that only replicated in the tonsils and respiratory tracts of ferrets^16^, CK/SD008 was detected in the tonsils throughout the lungs, and in the brains of the two ferrets, but not in the spleens or kidneys (Figure 3A).

We then evaluated the transmission of CK/SD008 in ferrets, a commonly used animal model for transmission studies^6,7,16,28^. We used the H7N9 virus CK/S1053, which did not transmit in our previous study^16^, as a negative control. Groups of three ferrets were i.n. inoculated with 10^6^ EID_50_ of CK/S1053 and CK/SD008, respectively. The ferrets were housed separately in cages within an isolator. Twenty-four hours later, three naive ferrets were placed in adjacent cages. Each pair of animals was separated by a divider as described previously^9,16,42,43^. Nasal washes were collected every 2 days from all of the animals beginning 2 days p.i. (1 day post-exposure (p.e.)) to assess virus shedding. Sera were collected from all animals on day 14 p.i. for hemagglutinin inhibition (HI) antibody detection. Respiratory droplet transmission was confirmed when virus was detected in the nasal washes and by seroconversion of the naive exposed animals at the end of the 2-week observation period.

Virus was detected in all directly infected ferrets (Figure 3D, 3E). A low virus titer was detected on day 9 p.e. from one of the three ferrets that was exposed to the CK/SD008-infected ferrets (Figure 3E); no virus was detected from any ferrets exposed to the CK/S1053-infected ferrets (Figure 3D). Ferrets exposed or infected with CK/SD008 experienced a 1.9%-8.2% weight loss, whereas ferrets directly infected with CK/S1053 showed a 4.0%-4.6% weight loss (Figure 3I, 3J; Table 2). Body temperature increases were detected in two ferrets of each directly infected group (Supplementary information, Figure S5). Seroconversion occurred in all of the animals directly infected with virus, and in one exposed animal in the CK/SD008 group (Table 2). Similar to our findings in 2013^16^, these results indicate that CK/SD008 transmits in ferrets by respiratory droplet with low efficiency.

### Replication and virulence of CK/SD008 PB2 mutants in mice

Two amino acids in PB2, 627K, and 701N are important for the virulence and transmission of influenza viruses in mammals^22,23,24,25,26,27^. We previously showed that some H9N2 viruses, which have similar internal genes to H7N9 viruses, readily acquire the PB2 627K or PB2 701N mutation upon infection of ferrets^42^. The CK/SD008 virus does not contain either of these two PB2 mutations. To investigate whether these mutations occurred in CK/SD008 during its replication in ferrets, we sequenced the PB2 gene of the viruses recovered from the organs of ferrets on day 4 p.i. and from the nasal washes of ferrets that participated in the transmission study. We found viruses bearing PB2 627K and viruses bearing PB2 701N in the organs (Supplementary information, Table S2) and nasal washes of ferrets that were directly infected in the transmission study (Supplementary information, Table S3). The virus in the nasal washes of the exposed animals contained only the PB2 627K mutation (Supplementary information, Table S3).

The PB2 627K and 701N mutations were not detected in any of the H7N9 viruses isolated from poultry (Table 1); however, about 83% of 648 H7N9 viruses isolated from humans contain PB2 627K or PB2 701N (Table 1), indicating that the H7N9 viruses could easily obtain these mutations during their replication in humans. To assess the risk of a CK/SD008-like virus obtaining such mutations after replicating in humans, we purified the mutants from the ferret lung samples by limited dilution in eggs. Two viruses were confirmed by genome sequence analysis to each contain one of the two PB2 mutations (CK/SD008-PB2/627K and CK/SD008-PB2/701N) and were then used for further studies in mice and ferrets.

As observed in other influenza viruses^44^, the PB2 627K and 701N mutations also significantly increased the viral polymerase activity of the CK/SD008 virus (Supplementary information, Figure S4). The viral titers of CK/SD008-PB2/627K and CK/SD008-PB2/701N in the nasal turbinates and lungs of mice were significantly higher than those of mice inoculated with CK/SD008. CK/SD008-PB2/627K was also detected in the brains of all three mice, and in the spleen and kidneys of one mouse (Figure 2C). Both viruses caused severe disease and killed mice at low doses (MLD_50_ values of CK/SD008-PB2/627K and CK/SD008-PB2/701N were 1.8log_10_EID_50_ and 3.4log_10_EID_50_, respectively) (Figure 2E, 2F, 2H, 2I).

### Replication, virulence, and transmission of CK/SD008 PB2 mutants in ferrets

We next evaluated the replication of the two viruses in ferrets. The tissue tropism and viral titers in the organs of ferrets inoculated with CK/SD008-PB2/627K and CK/SD008-PB2/701N were comparable with those of the ferrets inoculated with CK/SD008 (Figure 3). We then investigated the respiratory droplet transmission of CK/SD008-PB2/627K and CK/SD008-PB2/701N in ferrets. In the directly infected groups, virus was detected from all animals; in the CK/SD008-PB2/627K-exposed group, virus was detected in one ferret on day 1 p.e. and in all three animals on the other days tested (Figure 3F); in the CK/SD008-PB2/701N-exposed group, virus was detected in one ferret on days 3 and 5 p.e. and in two ferrets on day 7 and 9 p.e. (Figure 3G). One ferret infected with CK/SD008-PB2/627K had a body temperature increase on day 7 p.i., but no marked body temperature change was detected in the CK/SD008-PB2/701N-infected ferrets (Supplementary information, Figure S5). The ferrets directly infected with virus lost 11.3%-28.9% of their body weight (Figure 3K, 3L). One CK/SD008-PB2/627K-inoculated ferret was killed because it showed signs of severe disease, including tremor and torticollis, and became paralyzed on day 10 p.i.; one CK/SD008-PB2/701N-inoculated ferret became very sick and died on day 10 p.i. (Table 2). Seroconversion occurred in all of the inoculated animals and exposed animals (Table 2).

We repeated the ferret transmission study of the CK/SD008-PB2/627K virus, along with CK/S1053 and A/Anhui/1/2013 (AH/1) as negative and positive controls, respectively. Transmission of CK/S1053 was not detected, but CK/SD008-PB2/627K and AH/1 were transmitted to all three animals. Moreover, one CK/SD008-PB2/627K-inoculated ferret lost 26.9% of its body weight and died on day 8 p.i. (Figure 3D, 3F, 3H; Table 2). These results indicate that the newly emerged chicken-lethal H7N9 virus became highly lethal in mice and efficiently transmissible in ferrets after obtaining the 627K or 701N mutation in its PB2.

### Receptor-binding preference of the CK/SD008 virus

**Figure 4:**
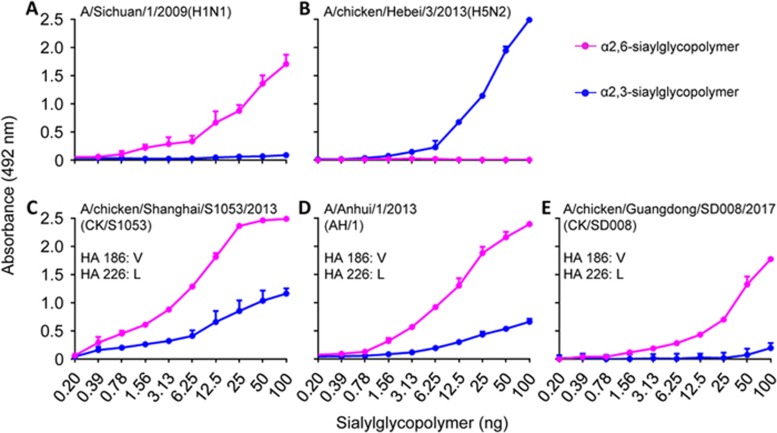
Receptor-binding properties of influenza viruses. Viruses were compared for their ability to bind to sialyglycopolymers containing either α2,6-siaylglycopolymer or α2,3-siaylglycopolymer.

Receptor-binding preference is important for influenza virus transmission, and two amino-acid mutations in HA, G186V, and Q226L, which play key roles in H7 avian influenza virus binding to human-type receptors^18^, appeared in over 90% of the H7N9 avian influenza field viruses we isolated in this study (Table 1). We tested the receptor-binding properties of the CK/SD008 and two viruses that were isolated in 2013, CK/S1053 and AH/1, which all bear HA 186V and 226L, and found that they all bound to the α2, 6-siaylglycopolymer (human-type receptor) with higher affinity than that of their binding with the α2, 3-siaylglycopolymer (avian-type receptor) (Figure 4).

### Thermal stability of H7N9 viruses

Thermal stability is also reported to be important for the transmissibility of some highly pathogenic H5N1 laboratory-adapted viruses^6^. We therefore compared the thermal stability of seven influenza viruses, including the five H7N9 viruses we used in the transmission study, a 2016 H7N9 avian virus, an H5N1 avian influenza virus, with the 2009 H1N1 human pandemic virus (SC/1), using the method described by Imai *et al*.^6^. We found that the two 2013 H7N9 viruses CK/S1053 and AH/1 were less stable than the human virus SC/1, but the CK/SD008 virus and its PB2 mutants, as well as the 2016 H7N9 virus and the H5N1 virus, were comparable to or more thermal stable than the human pandemic virus SC/1(Supplementary information, Figure S6).

## Discussion

Influenza viruses mutate; it is their nature. The most undesirable mutations are the ones that convert a low pathogenic avian influenza virus to a highly pathogenic avian influenza virus and the ones that allow a new influenza virus to be transmissible in humans. In this study, we show that these alarming mutations are occurring in the H7N9 viruses. Our findings provide important information for the control of H7N9 influenza. As soon as we detected the highly pathogenic H7N9 mutants in chickens, a series of actions were taken to prevent and minimize the damage they could cause to both poultry and humans, including the establishment of a rapid differential diagnostic test^45^, urgent surveillance in poultry, careful analysis of samples from human patients, and evaluation of a poultry vaccine.

Virulence and transmission of influenza viruses are polygenic traits. The MLD_50_ of CK/SD008-PB2/627K was 1.8log_10_EID_50_, indicating that this virus was > 10^5^-fold more lethal than CK/SD008 (MLD_50_, > 7.5log_10_EID_50_), and > 10^3^-fold more lethal than the 2013 H7N9 human isolates bearing the PB2 627K mutation, whose MLD_50_s were > 5.4log_10_EID_50_^16^. Therefore, the high virulence in mice of CK/SD008-PB2/627K represents genetic changes in both the PB2 and HA genes.

The virulence in mammals of these H7N9 PB2 mutants is similar to that of H5N1 highly pathogenic viruses that caused an ∼ 60% mortality rate in infected humans^46,47,48^, and is over 20% higher than that caused by the current low pathogenic H7N9 viruses in humans. Previous studies suggested that H5N1 mutants that were transmissible in ferrets were attenuated in mammals relative to their wild-type parent viruses, with the loss of virulence being the tradeoff for transmissibility^6,7^. We previously reported that, after replication in humans, low pathogenic H7N9 viruses could become transmissible in ferrets, but the viruses were not highly lethal in mice or ferrets^16^. In this study, we found that after the new H7N9 chicken-lethal virus acquired the PB2 627K or 701N mutation, it not only became highly transmissible in ferrets, but also became highly lethal in mice and caused severe disease and death in ferrets. Given that over 83% of the low pathogenic H7N9 viruses have obtained the PB2 mutations during their replication in humans (Table 1), it is highly likely that the chicken-lethal H7N9 virus will obtain similar mutations in their PB2 when they replicate in humans. Thus, it is very important to carefully monitor and evaluate the H7N9 human isolates to prevent such a virus from transmitting among humans by aerosol.

After we detected the chicken-lethal H7N9 viruses, we performed vital surveillance, collecting 2 950 samples from chicken farms and live poultry markets in Guangdong province in February, 2017. We isolated 28 H7N9 viruses (Supplementary information, Table S4), and partial sequence analysis of their HA genes revealed that 15 of them had the HA insertion (Supplementary information, Table S5). In addition to this insertion, we detected three additional motifs in the HA cleavage site of these viruses (Supplementary information, Table S5). The viruses with all of the other three different HA cleavage motifs were also lethal in chickens (Supplementary information, Table S5), although their virulence in mammals remains to be investigated. One of the motifs, -PKRKRTAR/G-, was found in isolates from patients in Guangdong province (Supplementary information, Table S5). The PB2 627K mutation was also detected in these human isolates (Supplementary information, Table S5).

We compared the thermal stability of different avian influenza viruses with the 2009 human pandemic H1N1 virus SC/1, and found that the naturally isolated duck H5N1 virus, the recent low and highly pathogenic H7N9 viruses, and the H7N9 PB2 mutants were all more thermally stable than the H1N1 pandemic virus. If thermal stability is important for influenza virus transmission, as reported by Imai *et al*.^6^, our results indicate that some of the H5N1 and H7N9 avian influenza viruses circulating in nature have already acquired this quality. The two H7N9 PB2 mutants are more thermally stable than the parent CK/SD008 virus, suggesting that similar to other properties of influenza virus, thermal stability may be a polygenic trait that is determined by a gene constellation rather than by the HA gene alone.

While this manuscript was in preparation, the H7N9 highly pathogenic virus has spread from Guangdong to several other provinces and caused huge outbreaks, and millions of chickens have been killed in efforts to control the disease^49^. It should be noted that, slaughtering the lethal H7N9 virus-infected poultry alone cannot solve this problem, because the broadly circulating low pathogenic H7N9 viruses can mutate to the highly pathogenic form at any time. Therefore, given the damage the H7N9 lethal virus will cause to poultry and the high risk it poses to human health, control and eradication of both the low and highly pathogenic H7N9 viruses should be the highest priority for animal disease control authorities in China.

## Materials and Methods

### Ethics statements

This study was carried out in strict accordance with the recommendations in the Guide for the Care and Use of Laboratory Animals of the Ministry of Science and Technology of the People's Republic of China. The protocols were approved by the Committee on the Ethics of Animal Experiments of the Harbin Veterinary Research Institute (HVRI) of the Chinese Academy of Agricultural Sciences (CAAS).

### Biosafety statement and facility

All experiments with live H7N9 viruses were conducted within the enhanced animal biosafety level 3 (ABSL3+) facility in the HVRI of the CAAS approved for such use by the Ministry of Agriculture of China. All animal studies were approved by the Review Board of the HVRI, CAAS. The details of the facility and the biosafety and biosecurity measures used have been previously reported^9^.

### Sample collection and virus isolation

From July 2013 to January 2017, 112 593 samples, including environment, cloacal, and tracheal swab samples of birds (cloacal and tracheal swabs of the same bird were put in the same sample collection tube and counted as one sample) from live poultry markets, poultry farms, poultry slaughterhouses, as well as faeces from wild bird habitats were collected in 24 provinces in China (Supplementary information, Table S1). Each sample was placed in 2 ml of minimal essential medium supplemented with penicillin (2 000 U/ml) and streptomycin (2 000 U/ml). All of the individual samples were inoculated into 10-day-old embryonated chicken eggs for 48 h at 37 °C. The allantoic fluid was collected and tested for HA activity with 0.5% chicken red blood cells. Where the HA assay was positive, HI assays were performed by using antisera against the 16 HA subtypes of avian influenza viruses and Newcastle disease virus (NDV), another avian virus frequently isolated from avian species. NA subtypes were determined by direct sequencing.

### Genetic and phylogenetic analysis

Viral RNA of H7N9 viruses was extracted from virus-infected allantoic fluid with the QIAmp viral RNA mini kit (Qiagen, Hilden, Germany). RT-PCR was performed with a set of gene-specific primers and the products were sequenced on an Applied Biosystems DNA analyzer. Primer sequences are available upon request. The nucleotide sequences were edited using the Seqman module of the DNAStar package. We performed the phylogenetic analysis using the Mega 6.0.6 ClustalW software package, implementing the neighbor-joining method. The tree topology was evaluated by 1 000 bootstrap analyses; 97% sequence identity cut-offs were used to categorize the groups of each gene segment in the phylogenetic trees.

We also created a Bayesian time-resolved phylogenetic tree for the HA gene of the group 1 and group 2 viruses using BEAST 1.8.4. The SRD06 nucleotide substitution model, the uncorrelated relaxed clock with a log-normal distribution, and the skygrid flexible effective population size tree prior were selected for the analysis. A Markov Chain Monte Carlo (MCMC) chain was run. The chain consisted of 30 000 000 steps and was sampled every 3 000 steps; the first 10% of samples were discarded as burn-in. The above MCMC settings were chosen to achieve a post burn-in effective sample size of at least 200 in all parameters, as recommended by the BEAST program^50^. The data were evaluated by using AICM in Tracer 1.6. The tree was viewed in Figtree 1.4.3.

### Animal studies

Randomization and blinding were not used for the allocation of animals to experimental groups.

### Chicken study

To determine the pathogenicity of the viruses, the IVPI was determined according to the recommendations of the Office International Des Epizooties^38^. Groups of ten 6-week-old SPF White Leghorn chickens housed in isolator cages were inoculated intravenously (i.v.) with 0.1 ml of a 1:10 dilution of bacterium-free allantoic fluid containing virus and were observed for signs of disease or death for 10 days. The organs, including brains, lungs, kidneys, spleens, pancreas, heart, liver, and cecum, of three dead birds inoculated with the index virus CK/SD008 were collected for virus titration in eggs.

Thirteen chickens were also inoculated i.n. with 10^6^ EID_50_ of CK/SD008 virus in a 0.1 ml volume, and three chickens were killed on day 3 p.i., and their tracheal and cloacal swabs, organs, including brains, lungs, kidneys, spleens, pancreas, heart, liver, and cecum were collected for virus titration in eggs. The remaining 10 birds and the negative control birds were observed for signs of disease or death for 10 days.

### Mouse study

To determine MLD_50_ values, groups of five mice (Vital River Laboratories, Beijing, China) were lightly anesthetized with CO_2_ and inoculated intranasally with 10-fold serial dilutions containing 10^1^-10^7^ 50% egg infectious doses (EID_50_) of CK/SD008 or 10^1^-10^6^ EID_50_ of CK/SD008-PB2/627K and CK/SD008-PB2/701N in a volume of 50 μl. The mice were monitored for 14 days for weight loss and mortality. To assess virus replication, groups of three mice were lightly anesthetized with CO_2_ and inoculated intranasally with 10^6^ EID_50_ of the test virus in a volume of 50 μl, and were then euthanized on day 3 p.i.; their nasal turbinates, lungs, spleens, kidneys, and brains were collected and titrated for virus infectivity in eggs.

### Ferret study

Four-month-old female ferrets (Wuxi Cay Ferret Farm, Jiangsu, China) that were serologically negative for influenza viruses were used in these studies. The animals were anesthetized via intramuscular injection with ketamine (20 mg/kg) and xylazine (1 mg/kg). To investigate virus replication, groups of two ferrets were anesthetized and inoculated i.n. with 10^6^ EID_50_ of test virus in a 500 l volume (250 μl per nostril). The ferrets were killed on day 4 p.i and the nasal turbinates, tonsils, trachea, lung, spleen, kidneys, and brain were collected for virus titration in eggs.

For the respiratory droplet transmission studies, groups of three ferrets were inoculated i.n. with 10^6^ EID_50_ of test virus and housed in specially designed cages inside an isolator as reported previously^9,16^. Twenty-four hours later, three naive animals were placed in an adjacent cage (4 cm away), separated by a double-layered net divider. These cages allow free passage of air. Nasal washes were collected at 2-day intervals, beginning on day 2 p.i. (1 day post exposure) and titrated in eggs. Sera were collected from all animals on day 14 p.i. for HI antibody detection. The ambient conditions for these studies were set at 20-22 °C and 30%-40% relative humidity. The airflow in the isolator was horizontal with a speed of 0.1 m/s; the airflow direction was from the inoculated animals to the exposed animals.

### Receptor-binding analysis

Receptor specificity was analysed by use of a solid-phase direct binding assay with two different glycopolymers: α-2, 3-siaylglycopolymer [Neu5Acα2-3Galß1-4GlcNAcß1-pAP (para-aminophenyl)-alpha-polyglutamic acid (α-PGA)] (avian-type receptor) and α-2, 6-sialylglycopolymer [Neu5Acα2-6Galß1-4GlcNAcß1-pAP (para-aminophenyl)-alpha-polyglutamic acid (a-PGA)] (human-type receptor) as described previously^9^. Chicken antisera against CK/S1053 virus, A/Sichuan/1/2009 (H1N1) virus, and A/chicken/Hebei/3/2013(H5N2) virus were generated in SPF chickens in our laboratory, and the horseradish peroxidase (HRP)-conjugated goat-anti-chicken antibody was purchased from Sigma-Aldrich (St. Louis, MO, USA).

### Heat stability test

Viruses (128 HA units in PBS) were incubated for the times indicated at 50 °C. Hemagglutination activity was then determined by use of hemagglutination assays using 0.5% chicken red blood cells, and infectivity was determined in 10-day-old chicken embryos.

### Polymerase activity analysis

A dual-luciferase reporter assay system (Promega) was used to compare the polymerase activities. Briefly, 0.5 μg of the firefly luciferase reporter plasmid p-Luci and the internal control plasmid Renilla were transfected into 293T cells together with 0.5 μg each of the four protein expression plasmids pCAGGS-PB2 (or pCAGGS-PB2/627K or pCAGGS-PB2/701N), pCAGGS-PB1, pCAGGS-PA, and pCAGGS-NP from the CK/SD008 virus. The assay was performed at 33 and 37 °C. Cell lysates were analysed 24 h after transfection to measure firefly and Renilla luciferase activities. Luminescence of the firefly luciferase was standardized using a plasmid expressing Renilla luciferase. Values shown are the mean ± SD of three independent experiments and are compared to those obtained with CK/SD008. The 293T cells purchased from ATCC have been tested to be mycoplasma negative by the commonly used PCR method.

### Statistical analysis

Virus titres of mice were statistically analysed by one-tailed paired *t*-test. The polymerase activity values were statistically analysed by two-tailed paired *t*-test.

## Author Contributions

JS, DG, ZB, and HC designed the study. JS, GD, HK, CG, SM, XY, XZ, PC, YC, HY, XW, XW, LL, PC, YJ, JL, YG, ML, ZQ, LG, JZ, WG, SH, YS, YH, ZW, LG, WY, LL, HB, GT, YL, CQ, and LJ performed the experiments. JS, CL, YS, ZB, and HC analysed the data. HC wrote the paper.

## Competing Financial Interests

The authors declare no competing financial interests.

## Figures and Tables

**Table 1:** Molecular characteristics of the HA and PB2 genes of the H7N9 viruses isolated from 2013-2017

Gene	Key molecular change		Avian isolates			Human isolates
			2013-2016	2017	Total	2013-2016
HA	Amino acids at the cleavage site	PKG ----R/G	253/253	33/40	286/293	648/648
		PKG***KRTA***R/G	0/253	7/40	7/293	0/648
	186V		240/253	40/40	280/293	638/648
	226L		235/253	31/40	266/293	620/648
PB2	627K		0/253	0/40	0/293	508/648
	701N		0/253	0/40	0/293	30/648

Sequences of H7N9 avian influenza viruses were obtained in this study from viruses that were isolated between July 2013 and January 2017; sequences of H7N9 human influenza viruses were obtained from the public database “Global Initiative on Sharing Avian Influenza Data”. The number on the left of the slash shows the number of viruses bearing the indicated mutations, and the number on the right of the slash shows the total number of viruses analyzed.

**Table 2:** Virulence and transmission of CK/SD008 and its mutants in ferrets

Virus	Maximum body temperature increase (°C)		Maximum body weight loss (%)		Survival/Total		Seroconversion (HI antibody titers)		Respiratory droplet transmission
	Inoculated	Exposed	Inoculated	Exposed	Inoculated	Exposed	Inoculated	Exposed	
CK/S1053^a^	2.6	0.5	4.6	4.0	6/6	6/6	6/6 (320–640)	0/6	None
CK/SD008	3.2	0.4	8.2	1.9	3/3	3/3	3/3 (160–640)	1/3 (80)	Inefficient
CK/SD008-PB2/627K^a^	2.0	0.8	26.9	7.6	4/6	6/6	4/4 (640–1280)	6/6 (160–1280)	Highly efficient
CK/SD008-PB2/701N	0.7	0.7	28.9	12.6	2/3	3/3	2/2 (320–640)	3/3 (160–640)	Highly efficient
AH/1	1.7	1.6	4.9	3.2	3/3	3/3	3/3 (320–640)	3/3 (160-640)	Highly efficient

Data shown are from the animal in that group with the maximum body temperature increase or maximum body weight loss. Seroconversion was confirmed from the sera of ferrets collected on day 14 post-infection.

^a^Each transmission test was conducted twice; the combined data from both experiments are shown.

## Supplementary Material

Supplementary information, Figure S1 Phylogenetic trees of the NA and internal genes of H7N9 influenza viruses.

Supplementary information, Figure S2 Bayesian time-resolved phylogenetic tree of the HA gene of H7N9 viruses.

Supplementary information, Figure S3 Geographic and temporal distribution of the different genotypes of H7N9 influenza viruses detected in this study.

Supplementary information, Figure S4 Polymerase activities of CK/SD008 and its PB2 mutants.

Supplementary information, Figure S5 Body temperature of ferrets after infection with or exposure to H7N9 viruses.

Supplementary information, Figure S6 Effect of heat-treatment on the hemagglutination activity and infectivity of influenza viruses.

Supplementary information, Table S1 Details of samples collected for H7N9 influenza virus isolation from July 2013 to January 2017^a^.

Supplementary information, Table S2 Amino acids at positions 627 and 701 of the PB2 gene of viruses recovered from the organs of ferrets that were inoculated with the CK/SD008 virus^a^.

Supplementary information, Table S3 Amino acids at positions 627 and 701 of the PB2 gene of viruses recovered from the nasal wash of ferrets that were inoculated with or exposed toCK/S1053 and CK/SD008.

Supplementary information, Table S4 Samples collected from Guangdong province in February 2017 and tested for H7N9 influenza viruses.

Supplementary information, Table S5 The amino acids in the cleavage site of HA and key amino acids in HA and PB2 related to receptor binding and virulence of H7N9 viruses isolated in 2017.
