# Supplementary material for: H7N9 virulent mutants detected in chickens in China pose an increased threat to humans
Source: Cell Res. 2017 Oct 24;27(12):1409–21. doi: 10.1038/cr.2017.129 (PMC5717404; doi:10.1038/cr.2017.129)
Supplement: Supplementary information, Figure S1 — Phylogenetic trees of the NA and internal genes of H7N9 influenza viruses. [file cr2017129x1.pdf]

Figure S1

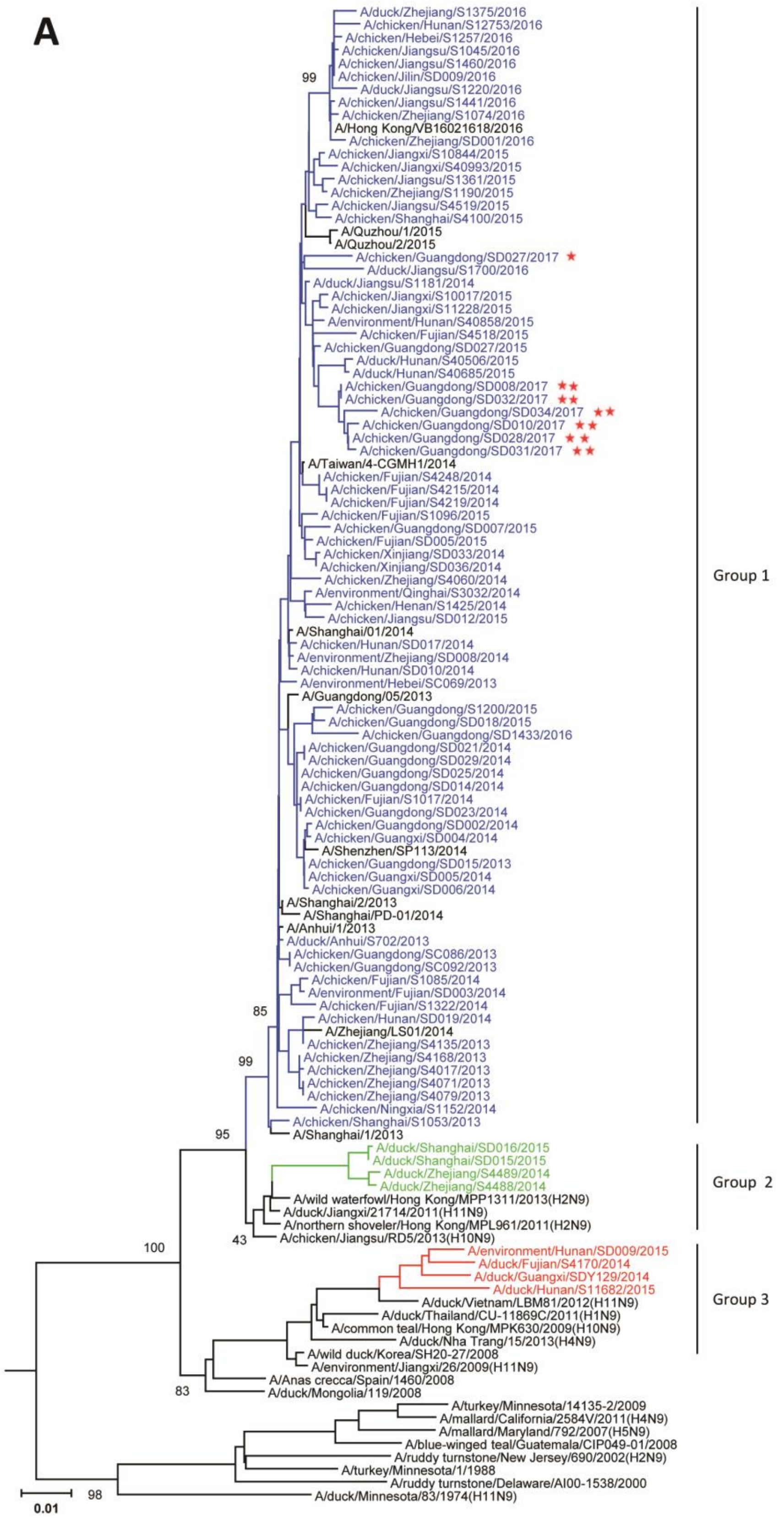
A
A/duck/Zhejiang/S1375/2016
A/chicken/Hunan/S12753/2016
A/chicken/Hebei/S1257/2016
A/chicken/Jiangsu/S1045/2016
A/chicken/Jiangsu/S1460/2016
A/chicken/Jilin/SD009/2016
A/duck/Jiangsu/S1220/2016
A/chicken/Jiangsu/S1441/2016
A/chicken/Zhejiang/S1074/2016
A/Hong Kong/VB16021618/2016
A/chicken/Zhejiang/SD001/2016
A/chicken/Jiangxi/S10844/2015
A/chicken/Jiangxi/S40993/2015
A/chicken/Jiangsu/S1361/2015
A/chicken/Zhejiang/S1190/2015
A/chicken/Jiangsu/S4519/2015
A/chicken/Shanghai/S4100/2015
A/Quzhou/1/2015
A/Quzhou/2/2015
A/chicken/Guangdong/SD027/2017 ★
A/duck/Jiangsu/S1700/2016
A/duck/Jiangsu/S1181/2014
A/chicken/Jiangxi/S10017/2015
A/chicken/Jiangxi/S11228/2015
A/environment/Hunan/S40858/2015
A/chicken/Fujian/S4518/2015
A/chicken/Guangdong/SD027/2015
A/duck/Hunan/S40506/2015
A/duck/Hunan/S40685/2015
A/chicken/Guangdong/SD008/2017 ★★
A/chicken/Guangdong/SD032/2017 ★★
A/chicken/Guangdong/SD034/2017 ★★
A/chicken/Guangdong/SD010/2017 ★★
A/chicken/Guangdong/SD028/2017 ★★
A/chicken/Guangdong/SD031/2017 ★★
A/Taiwan/4-CGMH1/2014
A/chicken/Fujian/S4248/2014
A/chicken/Fujian/S4215/2014
A/chicken/Fujian/S4219/2014
A/chicken/Fujian/S1096/2015
A/chicken/Guangdong/SD007/2015
A/chicken/Fujian/SD005/2015
A/chicken/Xinjiang/SD033/2014
A/chicken/Xinjiang/SD036/2014
A/chicken/Zhejiang/S4060/2014
A/environment/Qinghai/S3032/2014
A/chicken/Henan/S1425/2014
A/chicken/Jiangsu/SD012/2015
A/Shanghai/01/2014
A/chicken/Hunan/SD017/2014
A/environment/Zhejiang/SD008/2014
A/chicken/Hunan/SD010/2014
A/environment/Hebei/SC069/2013
A/Guangdong/05/2013
A/chicken/Guangdong/S1200/2015
A/chicken/Guangdong/SD018/2015
A/chicken/Guangdong/SD1433/2016
A/chicken/Guangdong/SD021/2014
A/chicken/Guangdong/SD029/2014
A/chicken/Guangdong/SD025/2014
A/chicken/Guangdong/SD014/2014
A/chicken/Fujian/S1017/2014
A/chicken/Guangdong/SD023/2014
A/chicken/Guangdong/SD002/2014
A/chicken/Guangxi/SD004/2014
A/Shenzhen/SP113/2014
A/chicken/Guangdong/SD015/2013
A/chicken/Guangxi/SD005/2014
A/chicken/Guangxi/SD006/2014
A/Shanghai/2/2013
A/Shanghai/PD-01/2014
A/Anhui/1/2013
A/duck/Anhui/S702/2013
A/chicken/Guangdong/SC086/2013
A/chicken/Guangdong/SC092/2013
A/chicken/Fujian/S1085/2014
A/environment/Fujian/SD003/2014
A/chicken/Fujian/S1322/2014
A/chicken/Hunan/SD019/2014
A/Zhejiang/LS01/2014
A/chicken/Zhejiang/S4135/2013
A/chicken/Zhejiang/S4168/2013
A/chicken/Zhejiang/S4017/2013
A/chicken/Zhejiang/S4071/2013
A/chicken/Zhejiang/S4079/2013
A/chicken/Ningxia/S1152/2014
A/chicken/Shanghai/S1053/2013
A/Shanghai/1/2013
A/duck/Shanghai/SD016/2015
A/duck/Shanghai/SD015/2015
A/duck/Zhejiang/S4489/2014
A/duck/Zhejiang/S4488/2014
A/wild waterfowl/Hong Kong/MPP1311/2013(H2N9)
A/duck/Jiangxi/21714/2011(H11N9)
A/northern shoveler/Hong Kong/MPL961/2011(H2N9)
A/chicken/Jiangsu/RD5/2013(H10N9)
A/environment/Hunan/SD009/2015
A/duck/Fujian/S4170/2014
A/duck/Guangxi/SDY129/2014
A/duck/Hunan/S11682/2015
A/duck/Vietnam/LBM81/2012(H11N9)
A/duck/Thailand/CU-11869C/2011(H1N9)
A/common teal/Hong Kong/MPK630/2009(H10N9)
A/duck/Nha Trang/15/2013(H4N9)
A/wild duck/Korea/SH20-27/2008
A/environment/Jiangxi/26/2009(H11N9)
A/Anas crecca/Spain/1460/2008
A/duck/Mongolia/119/2008
A/turkey/Minnesota/14135-2/2009
A/mallard/California/2584V/2011(H4N9)
A/mallard/Maryland/792/2007(H5N9)
A/blue-winged teal/Guatemala/CIP049-01/2008
A/ruddy turnstone/New Jersey/690/2002(H2N9)
A/turkey/Minnesota/1/1988
A/ruddy turnstone/Delaware/AI00-1538/2000
A/duck/Minnesota/83/1974(H11N9)
99
85
99
95
100
43
83
98
0.01
Group 1
Group 2
Group 3

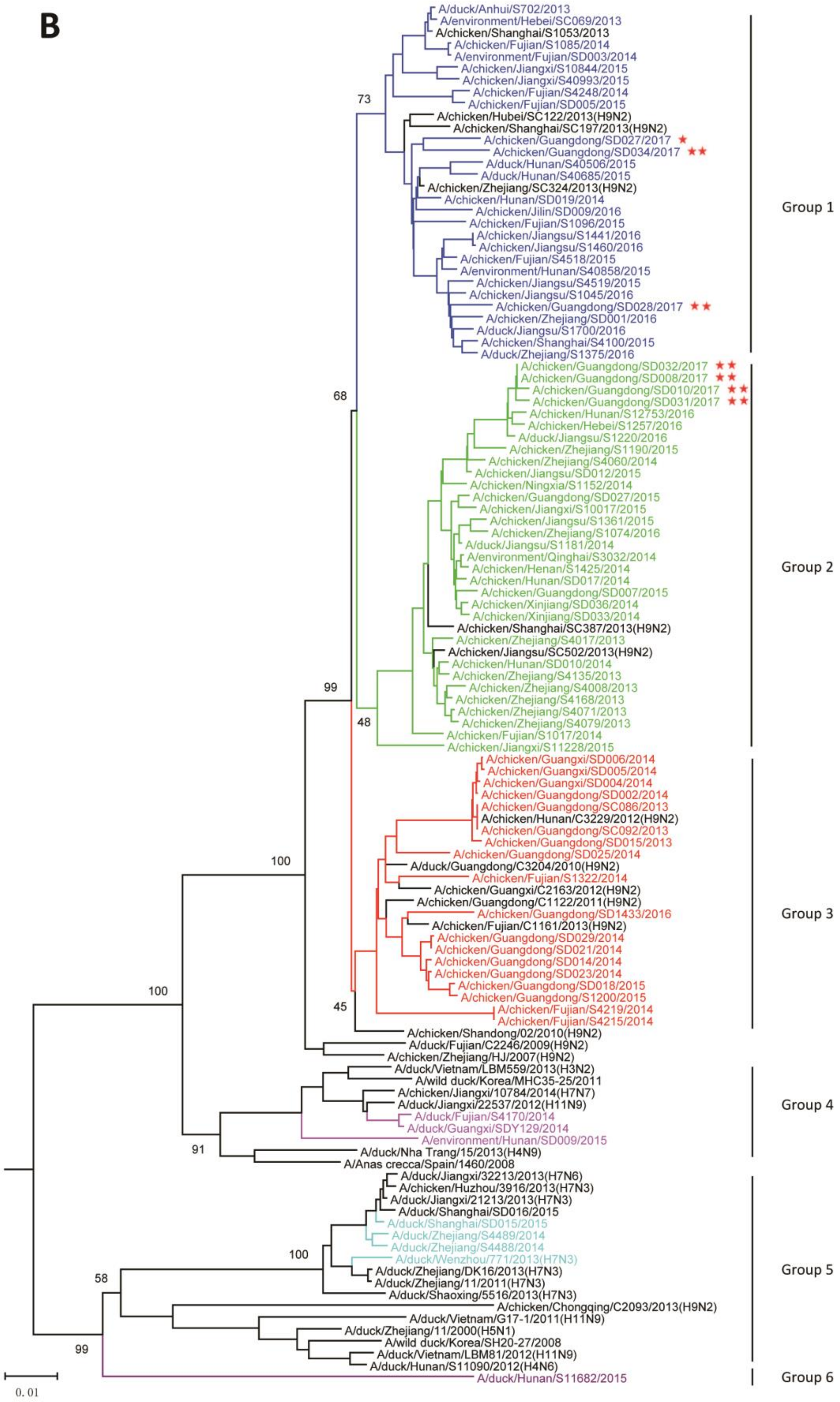
B
A/duck/Anhui/S702/2013
A/environment/Hebei/SC069/2013
A/chicken/Shanghai/S1053/2013
A/chicken/Fujian/S1085/2014
A/environment/Fujian/SD003/2014
A/chicken/Jiangxi/S10844/2015
A/chicken/Jiangxi/S40993/2015
A/chicken/Fujian/S4248/2014
A/chicken/Fujian/SD005/2015
A/chicken/Hubei/SC122/2013(H9N2)
A/chicken/Shanghai/SC197/2013(H9N2)
A/chicken/Guangdong/SD027/2017
A/chicken/Guangdong/SD034/2017
A/duck/Hunan/S40506/2015
A/duck/Hunan/S40685/2015
A/chicken/Zhejiang/SC324/2013(H9N2)
A/chicken/Hunan/SD019/2014
A/chicken/Jilin/SD009/2016
A/chicken/Fujian/S1096/2015
A/chicken/Jiangsu/S1441/2016
A/chicken/Jiangsu/S1460/2016
A/chicken/Fujian/S4518/2015
A/environment/Hunan/S40858/2015
A/chicken/Jiangsu/S4519/2015
A/chicken/Jiangsu/S1045/2016
A/chicken/Guangdong/SD028/2017
A/chicken/Zhejiang/SD001/2016
A/duck/Jiangsu/S1700/2016
A/chicken/Shanghai/S4100/2015
A/duck/Zhejiang/S1375/2016
Group 1
A/chicken/Guangdong/SD032/2017
A/chicken/Guangdong/SD008/2017
A/chicken/Guangdong/SD010/2017
A/chicken/Guangdong/SD031/2017
A/chicken/Hunan/S12753/2016
A/chicken/Hebei/S1257/2016
A/duck/Jiangsu/S1220/2016
A/chicken/Zhejiang/S1190/2015
A/chicken/Zhejiang/S4060/2014
A/chicken/Jiangsu/SD012/2015
A/chicken/Ningxia/S1152/2014
A/chicken/Guangdong/SD027/2015
A/chicken/Jiangxi/S10017/2015
A/chicken/Jiangsu/S1361/2015
A/chicken/Zhejiang/S1074/2016
A/duck/Jiangsu/S1181/2014
A/environment/Qinghai/S3032/2014
A/chicken/Henan/S1425/2014
A/chicken/Hunan/SD017/2014
A/chicken/Guangdong/SD007/2015
A/chicken/Xinjiang/SD036/2014
A/chicken/Xinjiang/SD033/2014
A/chicken/Shanghai/SC387/2013(H9N2)
A/chicken/Zhejiang/S4017/2013
A/chicken/Jiangsu/SC502/2013(H9N2)
A/chicken/Hunan/SD010/2014
A/chicken/Zhejiang/S4135/2013
A/chicken/Zhejiang/S4008/2013
A/chicken/Zhejiang/S4168/2013
A/chicken/Zhejiang/S4071/2013
A/chicken/Zhejiang/S4079/2013
A/chicken/Fujian/S1017/2014
A/chicken/Jiangxi/S11228/2015
Group 2
A/chicken/Guangxi/SD006/2014
A/chicken/Guangxi/SD005/2014
A/chicken/Guangxi/SD004/2014
A/chicken/Guangdong/SD002/2014
A/chicken/Guangdong/SC086/2013
A/chicken/Hunan/C3229/2012(H9N2)
A/chicken/Guangdong/SC092/2013
A/chicken/Guangdong/SD015/2013
A/chicken/Guangdong/SD025/2014
A/duck/Guangdong/C3204/2010(H9N2)
A/chicken/Fujian/S1322/2014
A/chicken/Guangxi/C2163/2012(H9N2)
A/chicken/Guangdong/C1122/2011(H9N2)
A/chicken/Guangdong/SD1433/2016
A/chicken/Fujian/C1161/2013(H9N2)
A/chicken/Guangdong/SD029/2014
A/chicken/Guangdong/SD021/2014
A/chicken/Guangdong/SD014/2014
A/chicken/Guangdong/SD023/2014
A/chicken/Guangdong/SD018/2015
A/chicken/Guangdong/S1200/2015
A/chicken/Fujian/S4219/2014
A/chicken/Fujian/S4215/2014
Group 3
A/chicken/Shandong/02/2010(H9N2)
A/duck/Fujian/C2246/2009(H9N2)
A/chicken/Zhejiang/HJ/2007(H9N2)
A/duck/Vietnam/LBM559/2013(H3N2)
A/wild duck/Korea/MHC35-25/2011
A/chicken/Jiangxi/10784/2014(H7N7)
A/duck/Jiangxi/22537/2012(H11N9)
A/duck/Fujian/S4170/2014
A/duck/Guangxi/SDY129/2014
A/environment/Hunan/SD009/2015
A/duck/Nha Trang/15/2013(H4N9)
A/Anas crecca/Spain/1460/2008
Group 4
A/duck/Jiangxi/32213/2013(H7N6)
A/chicken/Huzhou/3916/2013(H7N3)
A/duck/Jiangxi/21213/2013(H7N3)
A/duck/Shanghai/SD016/2015
A/duck/Shanghai/SD015/2015
A/duck/Zhejiang/S4489/2014
A/duck/Zhejiang/S4488/2014
A/duck/Wenzhou/771/2013(H7N3)
A/duck/Zhejiang/DK16/2013(H7N3)
A/duck/Zhejiang/11/2011(H7N3)
A/duck/Shaoxing/5516/2013(H7N3)
A/chicken/Chongqing/C2093/2013(H9N2)
A/duck/Vietnam/G17-1/2011(H11N9)
A/duck/Zhejiang/11/2000(H5N1)
A/wild duck/Korea/SH20-27/2008
A/duck/Vietnam/LBM81/2012(H11N9)
A/duck/Hunan/S11090/2012(H4N6)
Group 5
A/duck/Hunan/S11682/2015
Group 6
73
68
99
48
100
45
100
91
100
58
99
0. 01

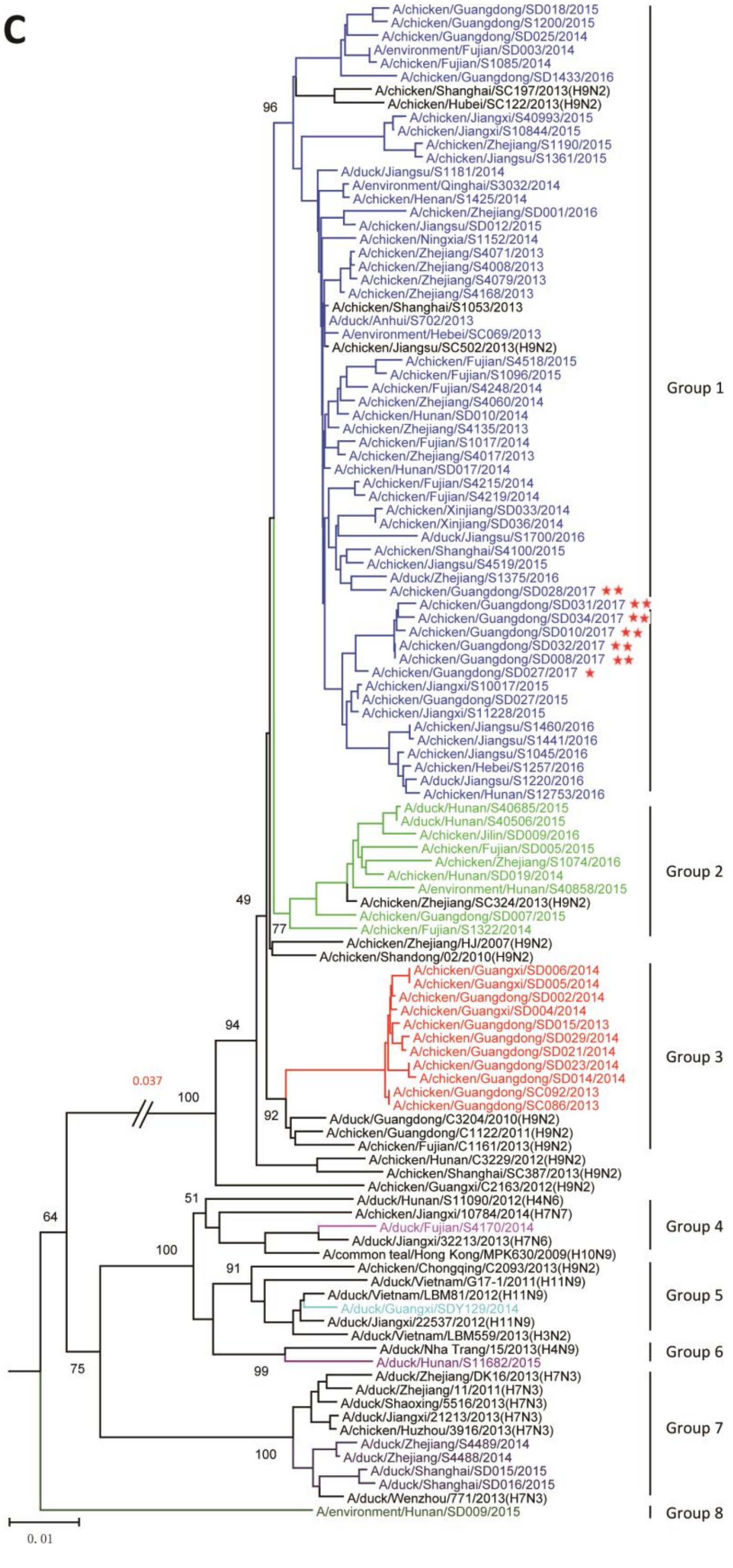
C
A/chicken/Guangdong/SD018/2015
A/chicken/Guangdong/S1200/2015
A/chicken/Guangdong/SD025/2014
A/environment/Fujian/SD003/2014
A/chicken/Fujian/S1085/2014
A/chicken/Guangdong/SD1433/2016
A/chicken/Shanghai/SC197/2013(H9N2)
A/chicken/Hubei/SC122/2013(H9N2)
96
A/chicken/Jiangxi/S40993/2015
A/chicken/Jiangxi/S10844/2015
A/chicken/Zhejiang/S1190/2015
A/chicken/Jiangsu/S1361/2015
A/duck/Jiangsu/S1181/2014
A/environment/Qinghai/S3032/2014
A/chicken/Henan/S1425/2014
A/chicken/Zhejiang/SD001/2016
A/chicken/Jiangsu/SD012/2015
A/chicken/Ningxia/S1152/2014
A/chicken/Zhejiang/S4071/2013
A/chicken/Zhejiang/S4008/2013
A/chicken/Zhejiang/S4079/2013
A/chicken/Zhejiang/S4168/2013
A/chicken/Shanghai/S1053/2013
A/duck/Anhui/S702/2013
A/environment/Hebei/SC069/2013
A/chicken/Jiangsu/SC502/2013(H9N2)
A/chicken/Fujian/S4518/2015
A/chicken/Fujian/S1096/2015
A/chicken/Fujian/S4248/2014
A/chicken/Zhejiang/S4060/2014
A/chicken/Hunan/SD010/2014
A/chicken/Zhejiang/S4135/2013
A/chicken/Fujian/S1017/2014
A/chicken/Zhejiang/S4017/2013
A/chicken/Hunan/SD017/2014
A/chicken/Fujian/S4215/2014
A/chicken/Fujian/S4219/2014
A/chicken/Xinjiang/SD033/2014
A/chicken/Xinjiang/SD036/2014
A/duck/Jiangsu/S1700/2016
A/chicken/Shanghai/S4100/2015
A/chicken/Jiangsu/S4519/2015
A/duck/Zhejiang/S1375/2016
A/chicken/Guangdong/SD028/2017 ★★
A/chicken/Guangdong/SD031/2017 ★★
A/chicken/Guangdong/SD034/2017 ★★
A/chicken/Guangdong/SD010/2017 ★★
A/chicken/Guangdong/SD032/2017 ★★
A/chicken/Guangdong/SD008/2017 ★★
A/chicken/Guangdong/SD027/2017 ★
A/chicken/Jiangxi/S10017/2015
A/chicken/Guangdong/SD027/2015
A/chicken/Jiangxi/S11228/2015
A/chicken/Jiangsu/S1460/2016
A/chicken/Jiangsu/S1441/2016
A/chicken/Jiangsu/S1045/2016
A/chicken/Hebei/S1257/2016
A/duck/Jiangsu/S1220/2016
A/chicken/Hunan/S12753/2016
Group 1
A/duck/Hunan/S40685/2015
A/duck/Hunan/S40506/2015
A/chicken/Jilin/SD009/2016
A/chicken/Fujian/SD005/2015
A/chicken/Zhejiang/S1074/2016
A/chicken/Hunan/SD019/2014
A/environment/Hunan/S40858/2015
A/chicken/Zhejiang/SC324/2013(H9N2)
A/chicken/Guangdong/SD007/2015
A/chicken/Fujian/S1322/2014
Group 2
49
77
A/chicken/Zhejiang/HJ/2007(H9N2)
A/chicken/Shandong/02/2010(H9N2)
A/chicken/Guangxi/SD006/2014
A/chicken/Guangxi/SD005/2014
A/chicken/Guangdong/SD002/2014
A/chicken/Guangxi/SD004/2014
A/chicken/Guangdong/SD015/2013
A/chicken/Guangdong/SD029/2014
A/chicken/Guangdong/SD021/2014
A/chicken/Guangdong/SD023/2014
A/chicken/Guangdong/SD014/2014
A/chicken/Guangdong/SC092/2013
A/chicken/Guangdong/SC086/2013
Group 3
94
0.037
100
92
A/duck/Guangdong/C3204/2010(H9N2)
A/chicken/Guangdong/C1122/2011(H9N2)
A/chicken/Fujian/C1161/2013(H9N2)
A/chicken/Hunan/C3229/2012(H9N2)
A/chicken/Shanghai/SC387/2013(H9N2)
A/chicken/Guangxi/C2163/2012(H9N2)
51
A/duck/Hunan/S11090/2012(H4N6)
64
A/chicken/Jiangxi/10784/2014(H7N7)
A/duck/Fujian/S4170/2014
A/duck/Jiangxi/32213/2013(H7N6)
A/common teal/Hong Kong/MPK630/2009(H10N9)
Group 4
100
91
A/chicken/Chongqing/C2093/2013(H9N2)
A/duck/Vietnam/G17-1/2011(H11N9)
A/duck/Vietnam/LBM81/2012(H11N9)
A/duck/Guangxi/SDY129/2014
A/duck/Jiangxi/22537/2012(H11N9)
A/duck/Vietnam/LBM559/2013(H3N2)
Group 5
A/duck/Nha Trang/15/2013(H4N9)
A/duck/Hunan/S11682/2015
Group 6
75
99
A/duck/Zhejiang/DK16/2013(H7N3)
A/duck/Zhejiang/11/2011(H7N3)
A/duck/Shaoxing/5516/2013(H7N3)
A/duck/Jiangxi/21213/2013(H7N3)
A/chicken/Huzhou/3916/2013(H7N3)
A/duck/Zhejiang/S4489/2014
A/duck/Zhejiang/S4488/2014
100
A/duck/Shanghai/SD015/2015
A/duck/Shanghai/SD016/2015
A/duck/Wenzhou/771/2013(H7N3)
Group 7
A/environment/Hunan/SD009/2015
Group 8
0. 01

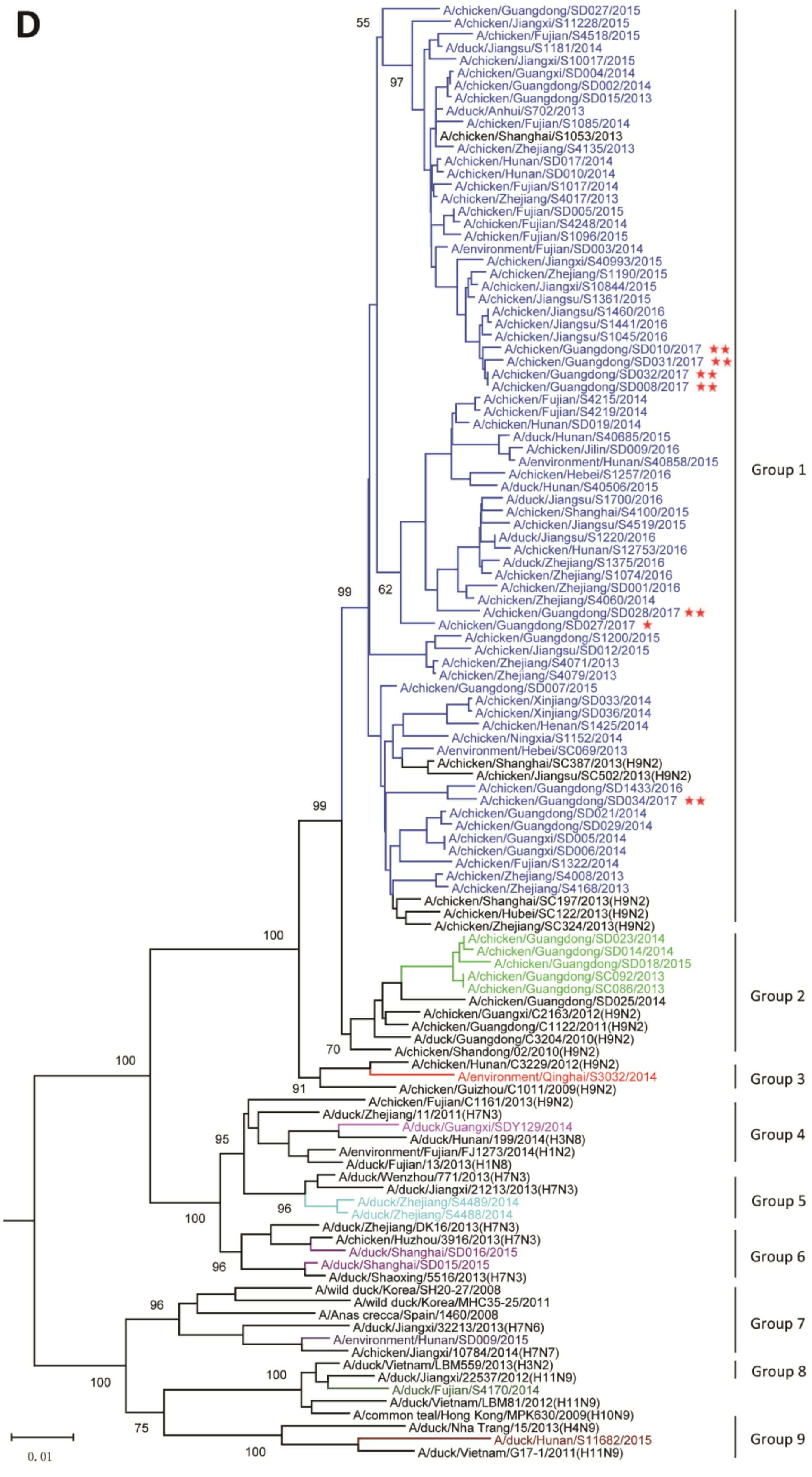
D
A/chicken/Guangdong/SD027/2015
A/chicken/Jiangxi/S11228/2015
A/chicken/Fujian/S4518/2015
A/duck/Jiangsu/S1181/2014
A/chicken/Jiangxi/S10017/2015
A/chicken/Guangxi/SD004/2014
A/chicken/Guangdong/SD002/2014
A/chicken/Guangdong/SD015/2013
A/duck/Anhui/S702/2013
A/chicken/Fujian/S1085/2014
A/chicken/Shanghai/S1053/2013
A/chicken/Zhejiang/S4135/2013
A/chicken/Hunan/SD017/2014
A/chicken/Hunan/SD010/2014
A/chicken/Fujian/S1017/2014
A/chicken/Zhejiang/S4017/2013
A/chicken/Fujian/SD005/2015
A/chicken/Fujian/S4248/2014
A/chicken/Fujian/S1096/2015
A/environment/Fujian/SD003/2014
A/chicken/Jiangxi/S40993/2015
A/chicken/Zhejiang/S1190/2015
A/chicken/Jiangxi/S10844/2015
A/chicken/Jiangsu/S1361/2015
A/chicken/Jiangsu/S1460/2016
A/chicken/Jiangsu/S1441/2016
A/chicken/Jiangsu/S1045/2016
A/chicken/Guangdong/SD010/2017 ★★
A/chicken/Guangdong/SD031/2017 ★★
A/chicken/Guangdong/SD032/2017 ★★
A/chicken/Guangdong/SD008/2017 ★★
A/chicken/Fujian/S4215/2014
A/chicken/Fujian/S4219/2014
A/chicken/Hunan/SD019/2014
A/duck/Hunan/S40685/2015
A/chicken/Jilin/SD009/2016
A/environment/Hunan/S40858/2015
A/chicken/Hebei/S1257/2016
A/duck/Hunan/S40506/2015
A/duck/Jiangsu/S1700/2016
A/chicken/Shanghai/S4100/2015
A/chicken/Jiangsu/S4519/2015
A/duck/Jiangsu/S1220/2016
A/chicken/Hunan/S12753/2016
A/duck/Zhejiang/S1375/2016
A/chicken/Zhejiang/S1074/2016
A/chicken/Zhejiang/SD001/2016
A/chicken/Zhejiang/S4060/2014
A/chicken/Guangdong/SD028/2017 ★★
A/chicken/Guangdong/SD027/2017 ★
A/chicken/Guangdong/S1200/2015
A/chicken/Jiangsu/SD012/2015
A/chicken/Zhejiang/S4071/2013
A/chicken/Zhejiang/S4079/2013
A/chicken/Guangdong/SD007/2015
A/chicken/Xinjiang/SD033/2014
A/chicken/Xinjiang/SD036/2014
A/chicken/Henan/S1425/2014
A/chicken/Ningxia/S1152/2014
A/environment/Hebei/SC069/2013
A/chicken/Shanghai/SC387/2013(H9N2)
A/chicken/Jiangsu/SC502/2013(H9N2)
A/chicken/Guangdong/SD1433/2016
A/chicken/Guangdong/SD034/2017 ★★
A/chicken/Guangdong/SD021/2014
A/chicken/Guangdong/SD029/2014
A/chicken/Guangxi/SD005/2014
A/chicken/Guangxi/SD006/2014
A/chicken/Fujian/S1322/2014
A/chicken/Zhejiang/S4008/2013
A/chicken/Zhejiang/S4168/2013
A/chicken/Shanghai/SC197/2013(H9N2)
A/chicken/Hubei/SC122/2013(H9N2)
A/chicken/Zhejiang/SC324/2013(H9N2)
Group 1
A/chicken/Guangdong/SD023/2014
A/chicken/Guangdong/SD014/2014
A/chicken/Guangdong/SD018/2015
A/chicken/Guangdong/SC092/2013
A/chicken/Guangdong/SC086/2013
A/chicken/Guangdong/SD025/2014
A/chicken/Guangxi/C2163/2012(H9N2)
A/chicken/Guangdong/C1122/2011(H9N2)
A/duck/Guangdong/C3204/2010(H9N2)
A/chicken/Shandong/02/2010(H9N2)
Group 2
A/chicken/Hunan/C3229/2012(H9N2)
A/environment/Qinghai/S3032/2014
A/chicken/Guizhou/C1011/2009(H9N2)
Group 3
A/chicken/Fujian/C1161/2013(H9N2)
A/duck/Zhejiang/11/2011(H7N3)
A/duck/Guangxi/SDY129/2014
A/duck/Hunan/199/2014(H3N8)
A/environment/Fujian/FJ1273/2014(H1N2)
A/duck/Fujian/13/2013(H1N8)
Group 4
A/duck/Wenzhou/771/2013(H7N3)
A/duck/Jiangxi/21213/2013(H7N3)
A/duck/Zhejiang/S4489/2014
A/duck/Zhejiang/S4488/2014
Group 5
A/duck/Zhejiang/DK16/2013(H7N3)
A/chicken/Huzhou/3916/2013(H7N3)
A/duck/Shanghai/SD016/2015
A/duck/Shanghai/SD015/2015
A/duck/Shaoxing/5516/2013(H7N3)
Group 6
A/wild duck/Korea/SH20-27/2008
A/wild duck/Korea/MHC35-25/2011
A/Anas crecca/Spain/1460/2008
A/duck/Jiangxi/32213/2013(H7N6)
A/environment/Hunan/SD009/2015
A/chicken/Jiangxi/10784/2014(H7N7)
Group 7
A/duck/Vietnam/LBM559/2013(H3N2)
A/duck/Jiangxi/22537/2012(H11N9)
A/duck/Fujian/S4170/2014
A/duck/Vietnam/LBM81/2012(H11N9)
A/common teal/Hong Kong/MPK630/2009(H10N9)
Group 8
A/duck/Nha Trang/15/2013(H4N9)
A/duck/Hunan/S11682/2015
A/duck/Vietnam/G17-1/2011(H11N9)
Group 9
55
97
99
62
99
100
100
70
91
95
96
100
96
96
100
100
75
100
0. 01

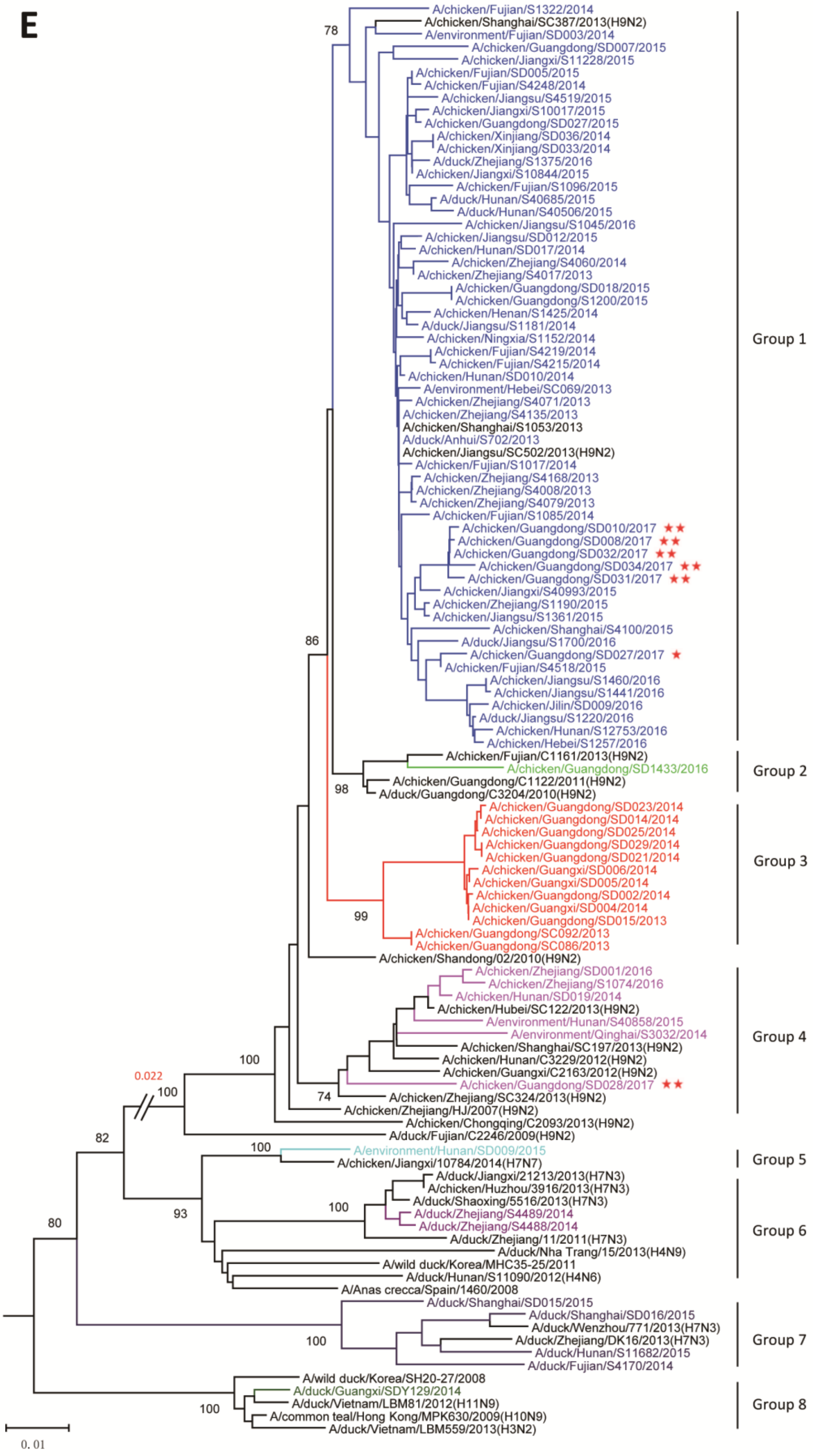
E
A/chicken/Fujian/S1322/2014
A/chicken/Shanghai/SC387/2013(H9N2)
A/environment/Fujian/SD003/2014
A/chicken/Guangdong/SD007/2015
A/chicken/Jiangxi/S11228/2015
A/chicken/Fujian/SD005/2015
A/chicken/Fujian/S4248/2014
A/chicken/Jiangsu/S4519/2015
A/chicken/Jiangxi/S10017/2015
A/chicken/Guangdong/SD027/2015
A/chicken/Xinjiang/SD036/2014
A/chicken/Xinjiang/SD033/2014
A/duck/Zhejiang/S1375/2016
A/chicken/Jiangxi/S10844/2015
A/chicken/Fujian/S1096/2015
A/duck/Hunan/S40685/2015
A/duck/Hunan/S40506/2015
A/chicken/Jiangsu/S1045/2016
A/chicken/Jiangsu/SD012/2015
A/chicken/Hunan/SD017/2014
A/chicken/Zhejiang/S4060/2014
A/chicken/Zhejiang/S4017/2013
A/chicken/Guangdong/SD018/2015
A/chicken/Guangdong/S1200/2015
A/chicken/Henan/S1425/2014
A/duck/Jiangsu/S1181/2014
A/chicken/Ningxia/S1152/2014
A/chicken/Fujian/S4219/2014
A/chicken/Fujian/S4215/2014
A/chicken/Hunan/SD010/2014
A/environment/Hebei/SC069/2013
A/chicken/Zhejiang/S4071/2013
A/chicken/Zhejiang/S4135/2013
A/chicken/Shanghai/S1053/2013
A/duck/Anhui/S702/2013
A/chicken/Jiangsu/SC502/2013(H9N2)
A/chicken/Fujian/S1017/2014
A/chicken/Zhejiang/S4168/2013
A/chicken/Zhejiang/S4008/2013
A/chicken/Zhejiang/S4079/2013
A/chicken/Fujian/S1085/2014
A/chicken/Guangdong/SD010/2017 ★★
A/chicken/Guangdong/SD008/2017 ★★
A/chicken/Guangdong/SD032/2017 ★★
A/chicken/Guangdong/SD034/2017 ★★
A/chicken/Guangdong/SD031/2017 ★★
A/chicken/Jiangxi/S40993/2015
A/chicken/Zhejiang/S1190/2015
A/chicken/Jiangsu/S1361/2015
A/chicken/Shanghai/S4100/2015
A/duck/Jiangsu/S1700/2016
A/chicken/Guangdong/SD027/2017 ★
A/chicken/Fujian/S4518/2015
A/chicken/Jiangsu/S1460/2016
A/chicken/Jiangsu/S1441/2016
A/chicken/Jilin/SD009/2016
A/duck/Jiangsu/S1220/2016
A/chicken/Hunan/S12753/2016
A/chicken/Hebei/S1257/2016
Group 1
A/chicken/Fujian/C1161/2013(H9N2)
A/chicken/Guangdong/SD1433/2016
A/chicken/Guangdong/C1122/2011(H9N2)
A/duck/Guangdong/C3204/2010(H9N2)
Group 2
A/chicken/Guangdong/SD023/2014
A/chicken/Guangdong/SD014/2014
A/chicken/Guangdong/SD025/2014
A/chicken/Guangdong/SD029/2014
A/chicken/Guangdong/SD021/2014
A/chicken/Guangxi/SD006/2014
A/chicken/Guangxi/SD005/2014
A/chicken/Guangdong/SD002/2014
A/chicken/Guangxi/SD004/2014
A/chicken/Guangdong/SD015/2013
A/chicken/Guangdong/SC092/2013
A/chicken/Guangdong/SC086/2013
Group 3
A/chicken/Shandong/02/2010(H9N2)
A/chicken/Zhejiang/SD001/2016
A/chicken/Zhejiang/S1074/2016
A/chicken/Hunan/SD019/2014
A/chicken/Hubei/SC122/2013(H9N2)
A/environment/Hunan/S40858/2015
A/environment/Qinghai/S3032/2014
A/chicken/Shanghai/SC197/2013(H9N2)
A/chicken/Hunan/C3229/2012(H9N2)
A/chicken/Guangxi/C2163/2012(H9N2)
A/chicken/Guangdong/SD028/2017 ★★
A/chicken/Zhejiang/SC324/2013(H9N2)
A/chicken/Zhejiang/HJ/2007(H9N2)
A/chicken/Chongqing/C2093/2013(H9N2)
A/duck/Fujian/C2246/2009(H9N2)
Group 4
A/environment/Hunan/SD009/2015
A/chicken/Jiangxi/10784/2014(H7N7)
Group 5
A/duck/Jiangxi/21213/2013(H7N3)
A/chicken/Huzhou/3916/2013(H7N3)
A/duck/Shaoxing/5516/2013(H7N3)
A/duck/Zhejiang/S4489/2014
A/duck/Zhejiang/S4488/2014
A/duck/Zhejiang/11/2011(H7N3)
A/duck/Nha Trang/15/2013(H4N9)
A/wild duck/Korea/MHC35-25/2011
A/duck/Hunan/S11090/2012(H4N6)
A/Anas crecca/Spain/1460/2008
Group 6
A/duck/Shanghai/SD015/2015
A/duck/Shanghai/SD016/2015
A/duck/Wenzhou/771/2013(H7N3)
A/duck/Zhejiang/DK16/2013(H7N3)
A/duck/Hunan/S11682/2015
A/duck/Fujian/S4170/2014
Group 7
A/wild duck/Korea/SH20-27/2008
A/duck/Guangxi/SDY129/2014
A/duck/Vietnam/LBM81/2012(H11N9)
A/common teal/Hong Kong/MPK630/2009(H10N9)
A/duck/Vietnam/LBM559/2013(H3N2)
Group 8
78
86
98
99
100
0.022
100
74
82
100
93
100
80
100
100
0. 01

F

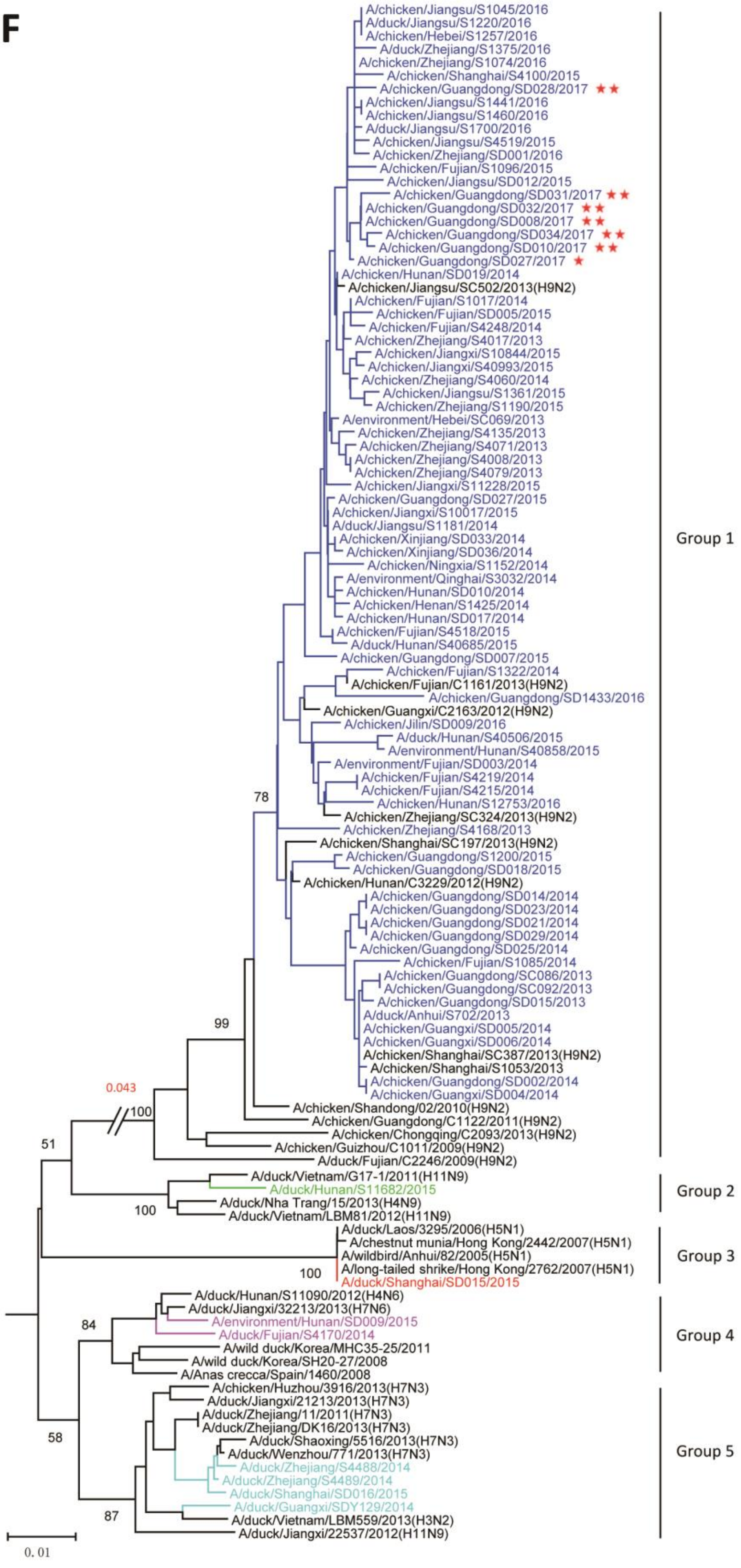

A/chicken/Jiangsu/S1045/2016
A/duck/Jiangsu/S1220/2016
A/chicken/Hebei/S1257/2016
A/duck/Zhejiang/S1375/2016
A/chicken/Zhejiang/S1074/2016
A/chicken/Shanghai/S4100/2015
A/chicken/Guangdong/SD028/2017 ★★
A/chicken/Jiangsu/S1441/2016
A/chicken/Jiangsu/S1460/2016
A/duck/Jiangsu/S1700/2016
A/chicken/Jiangsu/S4519/2015
A/chicken/Zhejiang/SD001/2016
A/chicken/Fujian/S1096/2015
A/chicken/Jiangsu/SD012/2015
A/chicken/Guangdong/SD031/2017 ★★
A/chicken/Guangdong/SD032/2017 ★★
A/chicken/Guangdong/SD008/2017 ★★
A/chicken/Guangdong/SD034/2017 ★★
A/chicken/Guangdong/SD010/2017 ★★
A/chicken/Guangdong/SD027/2017 ★
A/chicken/Hunan/SD019/2014
A/chicken/Jiangsu/SC502/2013(H9N2)
A/chicken/Fujian/S1017/2014
A/chicken/Fujian/SD005/2015
A/chicken/Fujian/S4248/2014
A/chicken/Zhejiang/S4017/2013
A/chicken/Jiangxi/S10844/2015
A/chicken/Jiangxi/S40993/2015
A/chicken/Zhejiang/S4060/2014
A/chicken/Jiangsu/S1361/2015
A/chicken/Zhejiang/S1190/2015
A/environment/Hebei/SC069/2013
A/chicken/Zhejiang/S4135/2013
A/chicken/Zhejiang/S4071/2013
A/chicken/Zhejiang/S4008/2013
A/chicken/Zhejiang/S4079/2013
A/chicken/Jiangxi/S11228/2015
A/chicken/Guangdong/SD027/2015
A/chicken/Jiangxi/S10017/2015
A/duck/Jiangsu/S1181/2014
A/chicken/Xinjiang/SD033/2014
A/chicken/Xinjiang/SD036/2014
A/chicken/Ningxia/S1152/2014
A/environment/Qinghai/S3032/2014
A/chicken/Hunan/SD010/2014
A/chicken/Henan/S1425/2014
A/chicken/Hunan/SD017/2014
A/chicken/Fujian/S4518/2015
A/duck/Hunan/S40685/2015
A/chicken/Guangdong/SD007/2015
A/chicken/Fujian/S1322/2014
A/chicken/Fujian/C1161/2013(H9N2)
A/chicken/Guangdong/SD1433/2016
A/chicken/Guangxi/C2163/2012(H9N2)
A/chicken/Jilin/SD009/2016
A/duck/Hunan/S40506/2015
A/environment/Hunan/S40858/2015
A/environment/Fujian/SD003/2014
A/chicken/Fujian/S4219/2014
A/chicken/Fujian/S4215/2014
A/chicken/Hunan/S12753/2016
A/chicken/Zhejiang/SC324/2013(H9N2)
78
A/chicken/Zhejiang/S4168/2013
A/chicken/Shanghai/SC197/2013(H9N2)
A/chicken/Guangdong/S1200/2015
A/chicken/Guangdong/SD018/2015
A/chicken/Hunan/C3229/2012(H9N2)
A/chicken/Guangdong/SD014/2014
A/chicken/Guangdong/SD023/2014
A/chicken/Guangdong/SD021/2014
A/chicken/Guangdong/SD029/2014
A/chicken/Guangdong/SD025/2014
A/chicken/Fujian/S1085/2014
A/chicken/Guangdong/SC086/2013
A/chicken/Guangdong/SC092/2013
A/chicken/Guangdong/SD015/2013
A/duck/Anhui/S702/2013
A/chicken/Guangxi/SD005/2014
A/chicken/Guangxi/SD006/2014
A/chicken/Shanghai/SC387/2013(H9N2)
A/chicken/Shanghai/S1053/2013
A/chicken/Guangdong/SD002/2014
A/chicken/Guangxi/SD004/2014
99
0.043
100
A/chicken/Shandong/02/2010(H9N2)
A/chicken/Guangdong/C1122/2011(H9N2)
A/chicken/Chongqing/C2093/2013(H9N2)
A/chicken/Guizhou/C1011/2009(H9N2)
51
A/duck/Fujian/C2246/2009(H9N2)
Group 1
A/duck/Vietnam/G17-1/2011(H11N9)
A/duck/Hunan/S11682/2015
A/duck/Nha Trang/15/2013(H4N9)
100
A/duck/Vietnam/LBM81/2012(H11N9)
Group 2
A/duck/Laos/3295/2006(H5N1)
A/chestnut munia/Hong Kong/2442/2007(H5N1)
A/wildbird/Anhui/82/2005(H5N1)
100
A/long-tailed shrike/Hong Kong/2762/2007(H5N1)
A/duck/Shanghai/SD015/2015
Group 3
A/duck/Hunan/S11090/2012(H4N6)
A/duck/Jiangxi/32213/2013(H7N6)
84
A/environment/Hunan/SD009/2015
A/duck/Fujian/S4170/2014
A/wild duck/Korea/MHC35-25/2011
A/wild duck/Korea/SH20-27/2008
A/Anas crecca/Spain/1460/2008
Group 4
A/chicken/Huzhou/3916/2013(H7N3)
A/duck/Jiangxi/21213/2013(H7N3)
A/duck/Zhejiang/11/2011(H7N3)
A/duck/Zhejiang/DK16/2013(H7N3)
58
A/duck/Shaoxing/5516/2013(H7N3)
A/duck/Wenzhou/771/2013(H7N3)
A/duck/Zhejiang/S4488/2014
A/duck/Zhejiang/S4489/2014
A/duck/Shanghai/SD016/2015
A/duck/Guangxi/SDY129/2014
87
A/duck/Vietnam/LBM559/2013(H3N2)
A/duck/Jiangxi/22537/2012(H11N9)
Group 5
0.01

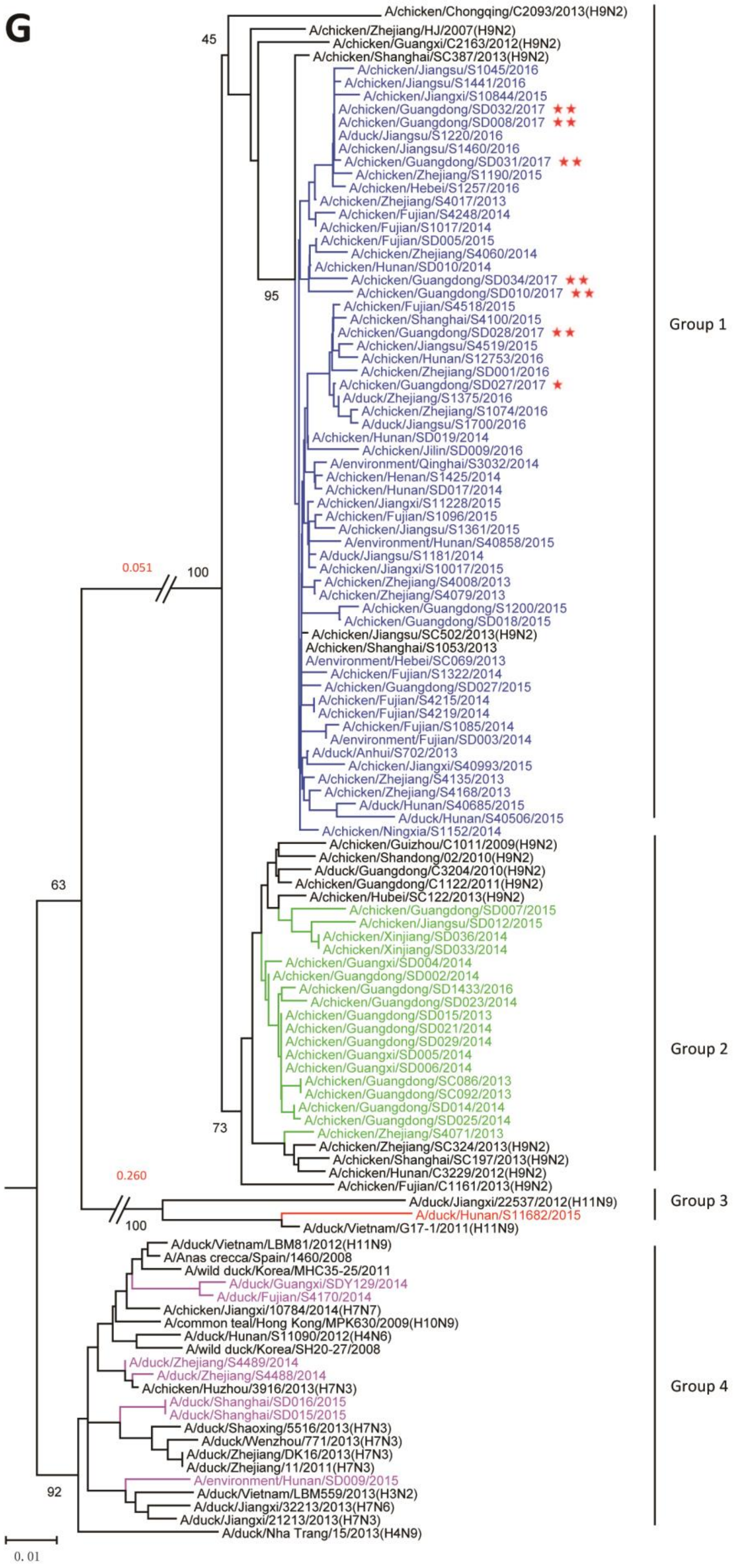

G
A/chicken/Chongqing/C2093/2013(H9N2)
A/chicken/Zhejiang/HJ/2007(H9N2)
A/chicken/Guangxi/C2163/2012(H9N2)
A/chicken/Shanghai/SC387/2013(H9N2)
A/chicken/Jiangsu/S1045/2016
A/chicken/Jiangsu/S1441/2016
A/chicken/Jiangxi/S10844/2015
A/chicken/Guangdong/SD032/2017
A/chicken/Guangdong/SD008/2017
A/duck/Jiangsu/S1220/2016
A/chicken/Jiangsu/S1460/2016
A/chicken/Guangdong/SD031/2017
A/chicken/Zhejiang/S1190/2015
A/chicken/Hebei/S1257/2016
A/chicken/Zhejiang/S4017/2013
A/chicken/Fujian/S4248/2014
A/chicken/Fujian/S1017/2014
A/chicken/Fujian/SD005/2015
A/chicken/Zhejiang/S4060/2014
A/chicken/Hunan/SD010/2014
A/chicken/Guangdong/SD034/2017
A/chicken/Guangdong/SD010/2017
A/chicken/Fujian/S4518/2015
A/chicken/Shanghai/S4100/2015
A/chicken/Guangdong/SD028/2017
A/chicken/Jiangsu/S4519/2015
A/chicken/Hunan/S12753/2016
A/chicken/Zhejiang/SD001/2016
A/chicken/Guangdong/SD027/2017
A/duck/Zhejiang/S1375/2016
A/chicken/Zhejiang/S1074/2016
A/duck/Jiangsu/S1700/2016
A/chicken/Hunan/SD019/2014
A/chicken/Jilin/SD009/2016
A/environment/Qinghai/S3032/2014
A/chicken/Henan/S1425/2014
A/chicken/Hunan/SD017/2014
A/chicken/Jiangxi/S11228/2015
A/chicken/Fujian/S1096/2015
A/chicken/Jiangsu/S1361/2015
A/environment/Hunan/S40858/2015
A/duck/Jiangsu/S1181/2014
A/chicken/Jiangxi/S10017/2015
A/chicken/Zhejiang/S4008/2013
A/chicken/Zhejiang/S4079/2013
A/chicken/Guangdong/S1200/2015
A/chicken/Guangdong/SD018/2015
A/chicken/Jiangsu/SC502/2013(H9N2)
A/chicken/Shanghai/S1053/2013
A/environment/Hebei/SC069/2013
A/chicken/Fujian/S1322/2014
A/chicken/Guangdong/SD027/2015
A/chicken/Fujian/S4215/2014
A/chicken/Fujian/S4219/2014
A/chicken/Fujian/S1085/2014
A/environment/Fujian/SD003/2014
A/duck/Anhui/S702/2013
A/chicken/Jiangxi/S40993/2015
A/chicken/Zhejiang/S4135/2013
A/chicken/Zhejiang/S4168/2013
A/duck/Hunan/S40685/2015
A/duck/Hunan/S40506/2015
A/chicken/Ningxia/S1152/2014
Group 1
45
95
100
0.051
63
A/chicken/Guizhou/C1011/2009(H9N2)
A/chicken/Shandong/02/2010(H9N2)
A/duck/Guangdong/C3204/2010(H9N2)
A/chicken/Guangdong/C1122/2011(H9N2)
A/chicken/Hubei/SC122/2013(H9N2)
A/chicken/Guangdong/SD007/2015
A/chicken/Jiangsu/SD012/2015
A/chicken/Xinjiang/SD036/2014
A/chicken/Xinjiang/SD033/2014
A/chicken/Guangxi/SD004/2014
A/chicken/Guangdong/SD002/2014
A/chicken/Guangdong/SD1433/2016
A/chicken/Guangdong/SD023/2014
A/chicken/Guangdong/SD015/2013
A/chicken/Guangdong/SD021/2014
A/chicken/Guangdong/SD029/2014
A/chicken/Guangxi/SD005/2014
A/chicken/Guangxi/SD006/2014
A/chicken/Guangdong/SC086/2013
A/chicken/Guangdong/SC092/2013
A/chicken/Guangdong/SD014/2014
A/chicken/Guangdong/SD025/2014
A/chicken/Zhejiang/S4071/2013
A/chicken/Zhejiang/SC324/2013(H9N2)
A/chicken/Shanghai/SC197/2013(H9N2)
A/chicken/Hunan/C3229/2012(H9N2)
A/chicken/Fujian/C1161/2013(H9N2)
73
Group 2
0.260
A/duck/Jiangxi/22537/2012(H11N9)
A/duck/Hunan/S11682/2015
A/duck/Vietnam/G17-1/2011(H11N9)
100
Group 3
A/duck/Vietnam/LBM81/2012(H11N9)
A/Anas crecca/Spain/1460/2008
A/wild duck/Korea/MHC35-25/2011
A/duck/Guangxi/SDY129/2014
A/duck/Fujian/S4170/2014
A/chicken/Jiangxi/10784/2014(H7N7)
A/common teal/Hong Kong/MPK630/2009(H10N9)
A/duck/Hunan/S11090/2012(H4N6)
A/wild duck/Korea/SH20-27/2008
A/duck/Zhejiang/S4489/2014
A/duck/Zhejiang/S4488/2014
A/chicken/Huzhou/3916/2013(H7N3)
A/duck/Shanghai/SD016/2015
A/duck/Shanghai/SD015/2015
A/duck/Shaoxing/5516/2013(H7N3)
A/duck/Wenzhou/771/2013(H7N3)
A/duck/Zhejiang/DK16/2013(H7N3)
A/duck/Zhejiang/11/2011(H7N3)
A/environment/Hunan/SD009/2015
A/duck/Vietnam/LBM559/2013(H3N2)
A/duck/Jiangxi/32213/2013(H7N6)
A/duck/Jiangxi/21213/2013(H7N3)
A/duck/Nha Trang/15/2013(H4N9)
92
Group 4
0. 01

Figure S1. Phylogenetic trees of the NA and internal genes of H7N9 influenza viruses. (**A**) Phylogenetic tree of NA. The tree was rooted to A/chicken/Italy/22A/1998(H5N9) virus and based on nucleotides (nt) 19 to 1416. The phylogenetic trees of PB2 (B), PB1(C), PA (D), NP (E), M (F), and NS (G) were based on nt 28–2307, 25–2298, 25–2175, 46–1542, 26–1007, and 27–864, respectively, and rooted to A/chicken/Rostock/45/1934(H7N1) virus. Sequences of viruses with names in black were downloaded from available databases; viruses with names in colors were sequenced in this study. Viruses labelled with stars contain the four amino acid (-KRTA-) insertion in their HA gene cleavage sites.
