# Supplementary material for: H7N9 virulent mutants detected in chickens in China pose an increased threat to humans
Source: Cell Res. 2017 Oct 24;27(12):1409–21. doi: 10.1038/cr.2017.129 (PMC5717404; doi:10.1038/cr.2017.129)
Supplement: Supplementary information, Figure S2 — Bayesian time-resolved phylogenetic tree of the HA gene of H7N9 viruses. [file cr2017129x2.pdf]

Figure S2

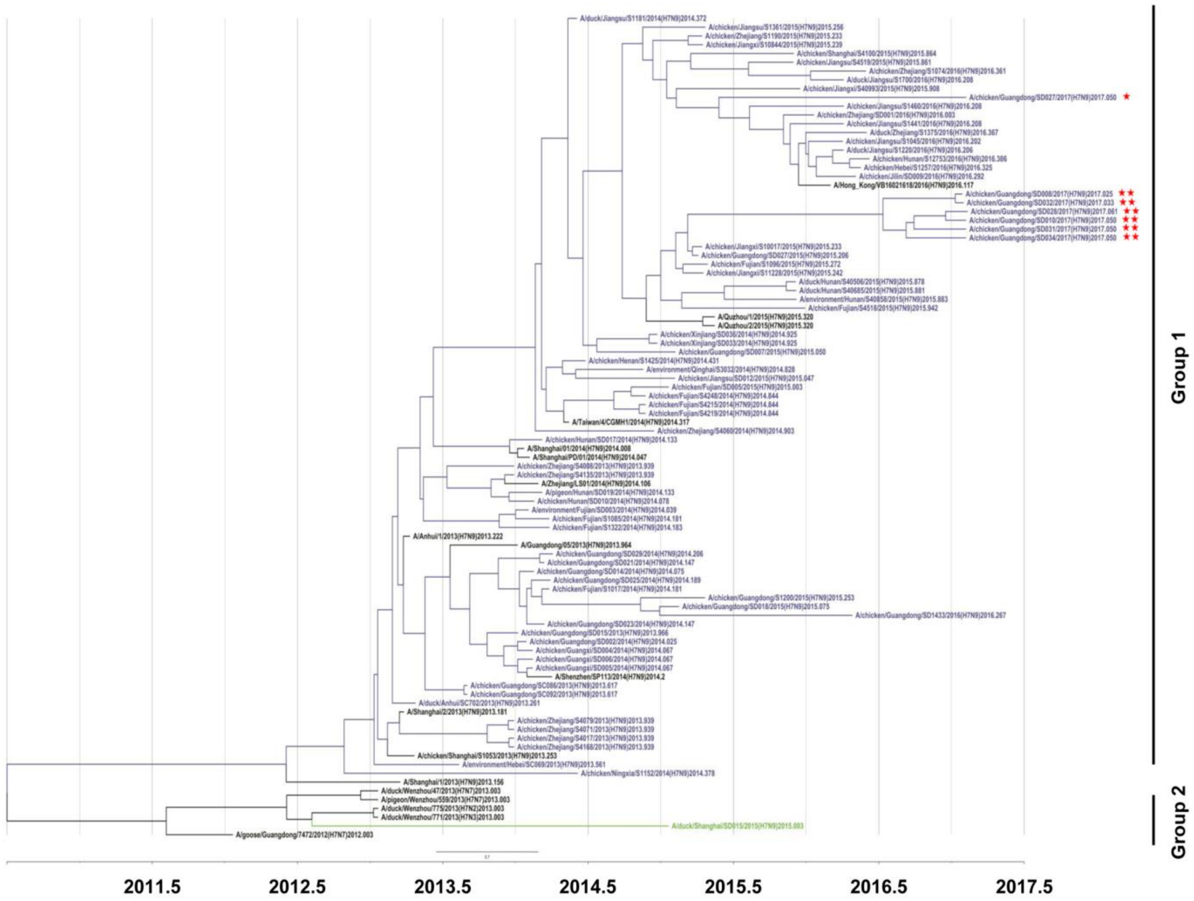

Figure S2. Bayesian time-resolved phylogenetic tree of the HA gene of H7N9 viruses. The tree was created using BEAST 1.8 with the SRD06 nucleotide substitution model, an uncorrelated relaxed clock with a log-normal distribution, and skygrid flexible effective population size tree prior. The tree was viewed in Figtree 1.4.3.
