# Supplementary material for: H7N9 virulent mutants detected in chickens in China pose an increased threat to humans
Source: Cell Res. 2017 Oct 24;27(12):1409–21. doi: 10.1038/cr.2017.129 (PMC5717404; doi:10.1038/cr.2017.129)
Supplement: Supplementary information, Figure S3 — Geographic and temporal distribution of the different genotypes of H7N9 influenza viruses detected in this study. [file cr2017129x3.pdf]

Figure S3

A

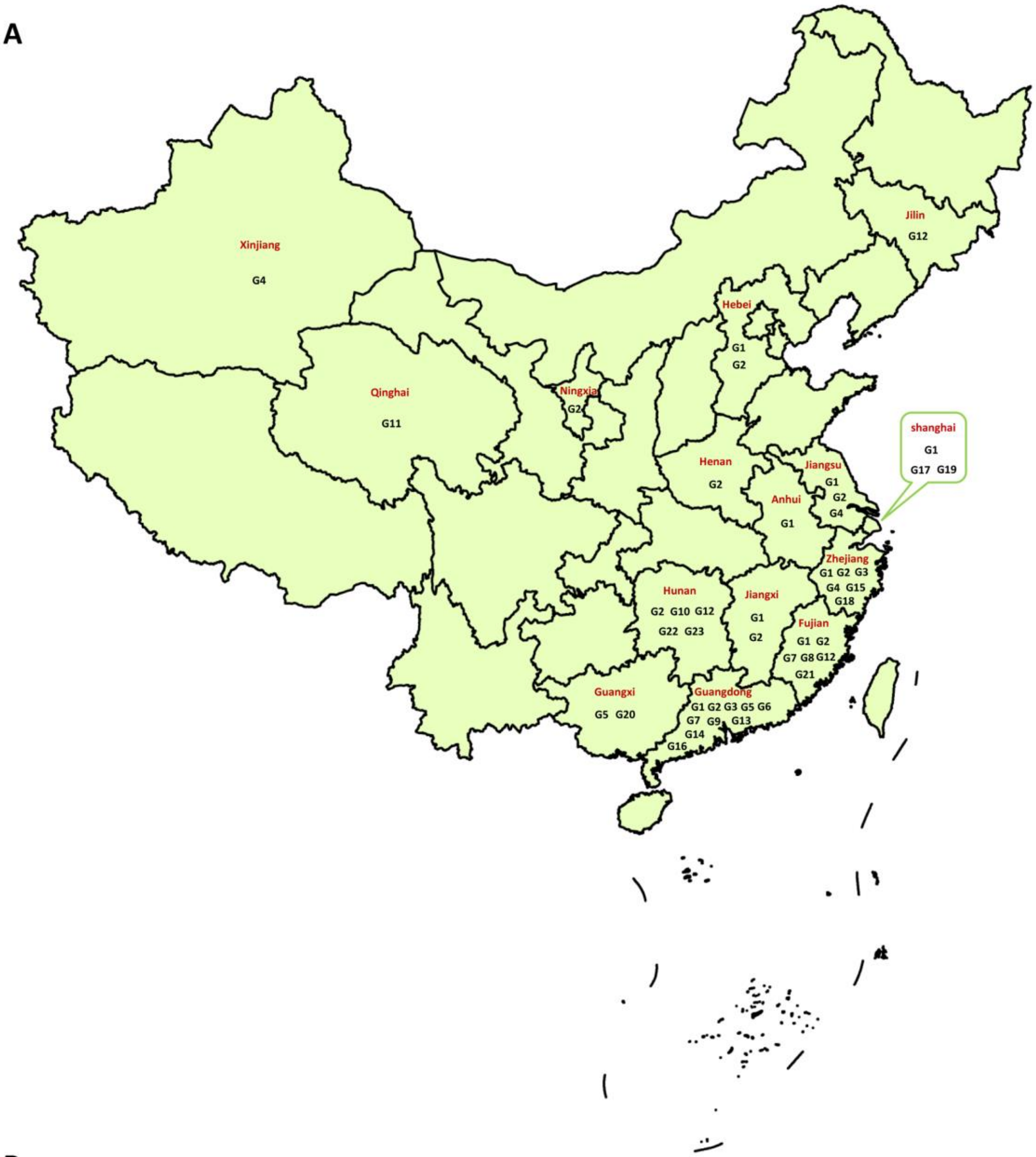

B

Year	Genotype
2013	G1, G2, G4, G5, G6
2014	G1, G2, G4, G5, G6, G7, G8, G9, G10, G11, G18, G20, G21
2015	G1, G2, G4, G7, G10, G12, G13, G14, G17, G19, G22, G23
2016	G1, G2, G3, G12, G15, G16
2017	G1, G2, G3

Figure S3. Geographic and temporal distribution of the different genotypes of H7N9 influenza viruses detected in this study.
