# Supplementary material for: H7N9 virulent mutants detected in chickens in China pose an increased threat to humans
Source: Cell Res. 2017 Oct 24;27(12):1409–21. doi: 10.1038/cr.2017.129 (PMC5717404; doi:10.1038/cr.2017.129)
Supplement: Supplementary information, Figure S4 — Polymerase activities of CK/SD008 and its PB2 mutants. [file cr2017129x4.pdf]

Figure S4

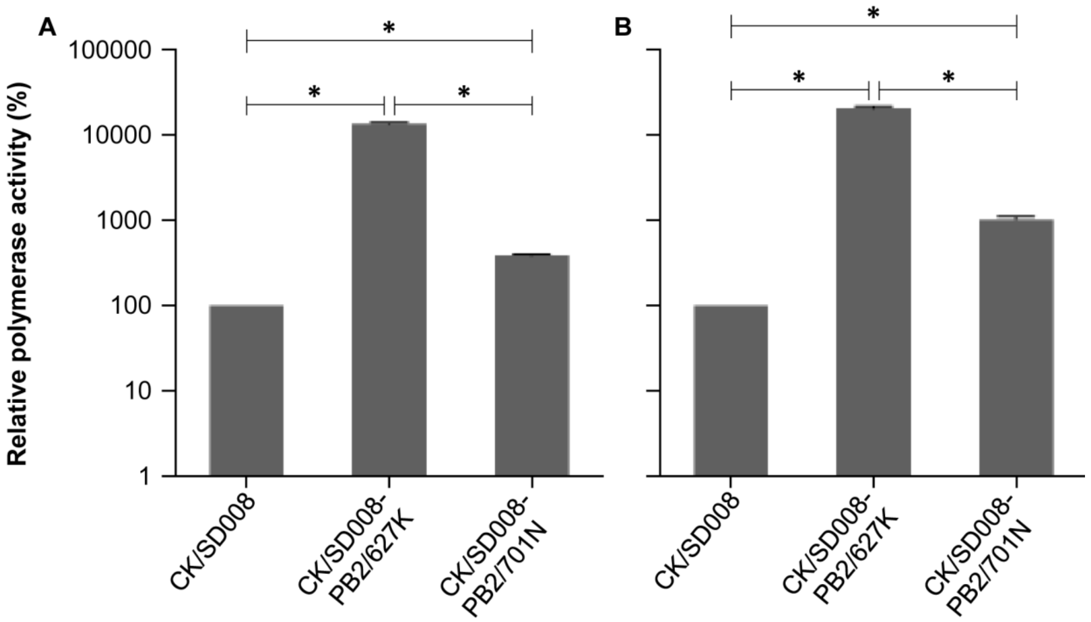

Figure S4. Polymerase activities of CK/SD008 and its PB2 mutants. 293T cells were transfected with plasmids containing PB2, PB1, PA, and NP genes of the indicated virus plus a firefly luciferase reporter plasmid. After transfection, the 293T cells were cultured at 33 ℃ (A) and 37 ℃ (B) for 24 h. Results are the averages from three independent experiments and are shown as the fold increase over the CK/SD008 virus value (100%). The values were statistically analyzed by using a two-tailed paired *t* test. *, $p < 0.01$.
