# Supplementary material for: H7N9 virulent mutants detected in chickens in China pose an increased threat to humans
Source: Cell Res. 2017 Oct 24;27(12):1409–21. doi: 10.1038/cr.2017.129 (PMC5717404; doi:10.1038/cr.2017.129)
Supplement: Supplementary information, Figure S5 — Body temperature of ferrets after infection with or exposure to H7N9 viruses. [file cr2017129x5.pdf]

Figure S5

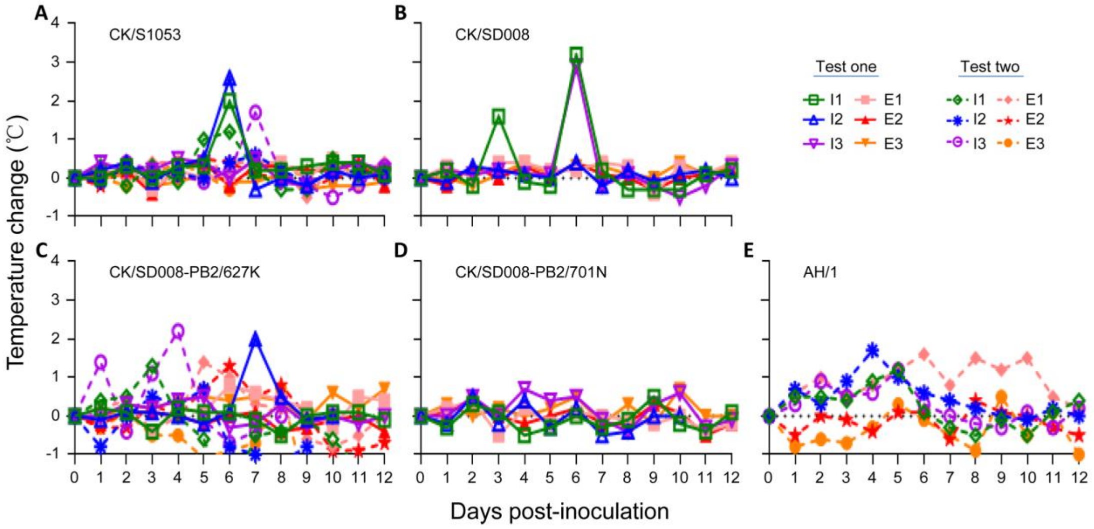

Figure S5. Body temperature of ferrets after infection with or exposure to H7N9 viruses. Change in body temperature in ferrets after infection with (I1-I3) or exposure to (E1-E3) CK/S1053 virus (A), CK/SD008 virus (B), CK/SD008-PB2/627K virus (C), CK/SD008-PB2/701N virus (D), and AH/1 virus (E). Changes were calculated by subtracting the mean temperature 3 days before infection from the temperature recorded on the indicated day.
