# Supplementary material for: H7N9 virulent mutants detected in chickens in China pose an increased threat to humans
Source: Cell Res. 2017 Oct 24;27(12):1409–21. doi: 10.1038/cr.2017.129 (PMC5717404; doi:10.1038/cr.2017.129)
Supplement: Supplementary information, Figure S6 — Effect of heat-treatment on the hemagglutination activity and infectivity of influenza viruses. [file cr2017129x6.pdf]

Figure S6

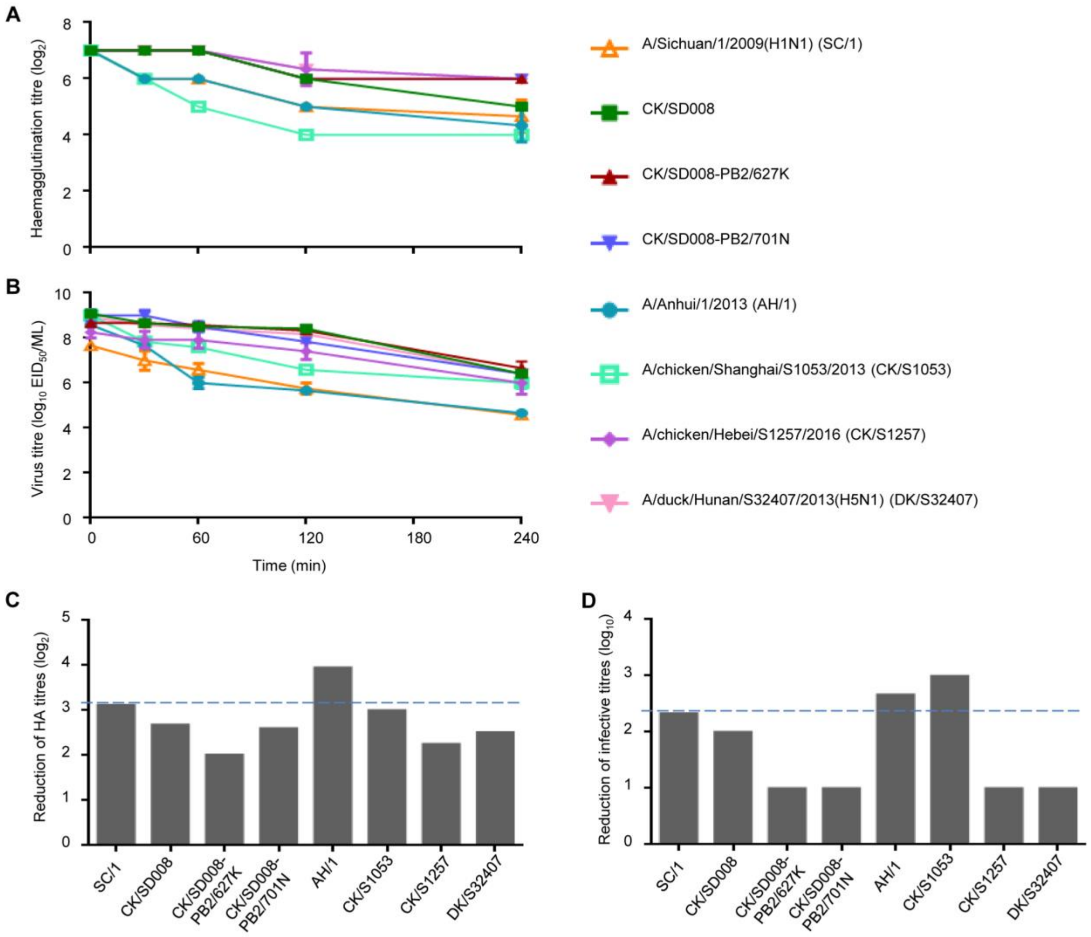

Figure S6. Effect of heat-treatment on the hemagglutination activity and infectivity of influenza viruses. Aliquots of viruses containing 128 HA units were incubated for the times indicated at 50 ℃. **A**. Hemagglutination titers in heat-treated samples were determined by using hemagglutination assay with 0.5% chicken red blood cells. **B**. Virus infectivity of heat-treated samples was determined in chicken eggs. Each point represents the mean ± standard deviation of triplicate experiments. **C**. Reduction in HA titers of each virus after 240 minutes of heat treatment. **D**. Reduction in viral infectivity of each virus after 240 minutes of heat treatment. The dashed lines indicate the reduced titer of human SC/1 virus after heat treatment for comparison.
