# Supplementary material for: H7N9 virulent mutants detected in chickens in China pose an increased threat to humans
Source: Cell Res. 2017 Oct 24;27(12):1409–21. doi: 10.1038/cr.2017.129 (PMC5717404; doi:10.1038/cr.2017.129)
Supplement: Supplementary information, Table S1 — Details of samples collected for H7N9 influenza virus isolation from July 2013 to January 2017a. [file cr2017129x7.pdf]

Table S1. Details of samples collected for H7N9 influenza virus isolation from July 2013 to January 2017[a].

Province or city	Sampling site	Samples collected (number of H7N9 viruses/number of total influenza viruses isolated)															
		2013			2014			2015			2016			2017			Total
		Chicken	Duck	Environ-ment	Chicken	Duck	Environ-ment	Chicken	Duck	Environ-ment	Chicken	Duck	Environ-ment	Chicken	Duck	Environ-ment	
Anhui	Poultry market	196 (0/15)	19 (0/2)	145 (0/6)	300 (0/3)	141 (0/19)	138 (0/11)	390 (5/16)	171 (2/27)	90 (0/5)	310 (3/19)	176 (2/34)	2 (0/1)				2,078 (12/158)
	Farm				410	10	40	180 (0/1)	133		270 (0/1)						1,043 (0/2)
Chong-qing	Poultry market	230 (0/9)	70 (0/9)	15	394 (0/17)	168 (0/23)	43 (0/3)	239 (0/20)	207 (0/24)	32 (0/8)	468 (0/18)	341 (0/38)	1 (0/1)				2,208 (0/170)
	Farm				216 (0/2)	82 (0/2)	18	330	30 (0/1)	10 (0/1)							686 (0/6)
Fujian	Poultry market	110 (0/6)	110 (0/12)	80 (0/12)	220 (4/5)	280 (1/19)	60 (1/5)	300 (4/10)	269 (0/30)	60 (2/14)	350 (4/17)	308 (0/28)	40 (0/4)				2,187 (16/162)
	Farm				30	120	10 (1/1)										160 (1/1)
Guang-dong	Poultry market	390 (4/9)	220 (0/14)	208 (0/7)	299 (11/31)	184 (0/24)	160 (0/15)	596 (34/67)	264 (0/32)	157 (0/15)	490 (7/27)	300 (1/28)	130 (0/12)	630 (31/51)			4,028 (88/332)
	Farm	140 (0/10)			219 (1/2)	43		85 (1/3)	30		30 (1/1)						547 (3/16)
Guangxi	Poultry market	380 (0/23)	260 (0/25)	100 (0/13)	897 (4/34)	1157 (0/82)	368 (0/22)	648 (0/34)	718 (0/92)	183 (0/26)	755 (0/29)	923 (0/98)	328 (0/35)				6,717 (4/513)
	Farm	30	150 (0/1)		310 (0/2)	494 (0/8)	10	430 (0/5)	470 (0/6)	20	480 (0/1)	540 (0/12)					2,934 (0/35)
Guizhou	Poultry market	330 (0/21)	60 (0/8)	90 (0/12)	491 (0/37)	86 (0/13)	40 (0/5)	560 (0/27)	192 (0/23)	130 (0/21)	620 (0/31)	194 (0/32)	52 (0/8)	42 (2/4)	23		2,910 (2/242)
	Farm		73	10	90				90 (0/1)								263 (0/1)
Hebei	Poultry market	70 (8/8)	4	40 (2/2)	40 (0/1)			127 (0/4)	50 (0/2)		359 (6/20)	53 (0/2)	40 (0/1)				783 (16/40)
	Farm	280			40	180		351 (0/1)		4							855 (0/1)
	Slaughter-house				350		50	30	60 (0/1)	20 (0/1)	100 (0/1)						610 (0/3)
Henan	Poultry	60		20	150			256	13	15	370	5	69				958

	market				(2/3)			(0/2)		(0/1)	(0/19)	(0/1)	(0/6)				(2/32)
	Farm	470 (0/1)		50	750			776		6	610 (0/1)						2,662 (0/2)
	Slaughter -house				120		40				60		30				250
Hubei	Poultry market	160 (0/8)	70 (0/7)	80 (0/5)	362 (0/14)	251 (0/37)	118 (0/16)	260 (5/19)	70 (0/6)	75 (0/5)	391 (0/8)	257 (0/18)	39 (0/3)				2,133 (5/146)
	Farm				70	538 (0/6)	112 (0/1)	30	78	10	60	300 (0/4)					1,198 (0/11)
	Slaughter -house										30						30
Hunan	Poultry market	120 (0/8)	100 (0/11)	90 (0/8)	531 (7/20)	262 (1/29)	163 (0/10)	535 (4/30)	439 (5/55)	150 (4/21)	475 (2/30)	448 (0/23)	141 (0/15)	30 (1/3)	10 (1/2)	20	3514 (25/265)
	Farm		1590 (0/10)	510 (0/12)	180	3137 (0/13)	1104 (0/23)		3104 (0/27)	790 (0/24)		3170 (0/22)	1000 (0/15)				14,585 (0/146)
Jiangsu	Poultry market	160 (0/9)	20 (0/4)	20 (0/3)	321 (7/19)	79 (3/16)	63 (1/6)	585 (4/28)	112 (1/17)	110 (1/14)	656 (21/44)	147 (1/18)	123(4/21)	60 (1/6)			2456 (44/205)
	Farm	180		10	347 (0/1)	90	158	300 (1/1)	270 (0/4)	20	200 (0/1)	90	15				1680 (1/7)
Jiangxi	Poultry market				299 (0/8)	319 (0/17)	70 (0/1)	256 (7/39)	210 (0/35)	81 (0/26)	408 (0/22)	195 (0/30)	25 (0/2)				1,863 (7/180)
	Farm		1218 (0/13)	360 (0/16)		1796 (0/21)	320 (0/9)		1317 (0/16)		120 (0/1)	363 (0/7)	50				5,544 (0/83)
Jilin	Poultry market	65		20	138 (0/1)	12 (0/2)					60 (2/2)						295 (2/5)
	Farm	300			1014	10		870			800						2,994
	Slaughter -house	30 (0/1)		20 (0/1)	60			300			120	30					560 (0/2)
Liaoning	Farm	60	210		750 (0/2)	150	30	890 (0/1)	100 (0/1)		780	227					3,197 (0/4)
	Slaughter -house	300	30	60 (0/1)	360 (0/1)	150	40	120			180						1,240 (0/2)
Ningxia	Poultry market	178 (0/14)	21 (0/3)	41 (0/3)	366 (1/22)	30 (0/2)	25	265 (0/6)	18	16 (0/1)	460 (0/10)	47 (0/1)	4				1,471 (1/62)
	Farm				360 (0/2)			270 (0/1)									630 (0/3)

	Slaughter<br>-house				90<br>(0/2)		30<br>(0/1)	30			30		11				191<br>(0/3)
Qinghai	Poultry<br>market				384<br>(0/8)	25<br>(0/4)	69<br>(1/1)	589<br>(0/17)	22	10<br>(0/2)	249<br>(0/3)						1348<br>(1/35)
	Farm				185<br>(0/2)		40				410<br>(0/1)						635<br>(0/3)
Shanxi	Farm	510<br>(0/1)			1200			1012<br>(0/1)		8	1020						3,750<br>(0/2)
Shandong	Poultry<br>market	155<br>(0/5)	3	98<br>(0/2)	30		3	255<br>(0/5)	14<br>(0/1)	45<br>(0/1)	240<br>(0/5)	40<br>(0/1)					883<br>(0/20)
	Farm	240			339	639<br>(0/2)	67<br>(0/1)	630<br>(0/6)	90<br>(0/2)	20<br>(0/1)	215	160	10				2,410<br>(0/12)
	Slaughter<br>-house							30	30	20	75<br>(0/1)	20					175<br>(0/1)
Shanghai	Poultry<br>market	190<br>(0/12)	10					150<br>(2/12)	30<br>(2/2)	30<br>(2/7)	370<br>(0/3)		30<br>(1/4)				810<br>(7/40)
	Farm							180									180
Sichuan	Poultry<br>market							269<br>(0/8)	177<br>(0/14)	28<br>(0/4)	517<br>(0/33)	282<br>(0/36)	30<br>(0/5)				1,303<br>(0/100)
	Farm				37	22	12	40	120	15							246
	Slaughter<br>-house								30<br>(0/2)								30<br>(0/2)
Tibet	Poultry<br>market				220<br>(0/9)	95<br>(0/7)	60<br>(0/6)	153	76<br>(0/2)	40<br>(0/1)	186<br>(0/8)	160<br>(0/13)	95<br>(0/7)				1,085<br>(0/53)
	Farm				170	50		320<br>(0/1)	16	10	40		10				616<br>(0/1)
Xinjiang	Poultry<br>market	151<br>(0/17)	44<br>(0/5)	50<br>(0/4)	220<br>(2/6)	23<br>(0/1)	31<br>(2/2)	286<br>(0/12)	59<br>(0/2)	49<br>(0/2)	390<br>(0/7)	27<br>(0/2)	60<br>(0/4)				1,390<br>(4/64)
	Farm	126	95<br>(0/1)	20	350<br>(0/1)		10	120<br>(0/7)	20<br>(0/2)	5	120	30					896<br>(0/11)
	Slaughter<br>house	95	15		111		30	96<br>(0/1)		10			30<br>(0/1)				387<br>(0/2)
Yunnan	Poultry<br>market				560<br>(0/9)	60<br>(0/6)	60<br>(0/7)	660<br>(0/18)	162<br>(0/12)	90<br>(0/4)	892<br>(0/31)	179<br>(0/11)	45<br>(0/1)				2,708<br>(0/99)
	Farm				60		10										70
Zhejiang	Poultry	185	116	145	243	57	170	250	116	34	480	284	68	60		50	2,258

	market	(12/14)	(0/1)	(6/10)	(6/27)	(1/11)	(1/18)	(2/8)	(2/19)	(1/4)	(11/28)	(2/34)	(3/10)	(1/4)		(3/7)	(51/195)
	Farm				270	360<br>(0/8)	100<br>(0/4)		840<br>(0/11)		30<br>(1/1)						1600<br>(1/24)
Total		5,891<br>(24/191)	4508<br>(0/126)	2,282<br>(8/117)	14,953<br>(45/291)	11,100<br>(6/372)	3,872<br>(7/168)	15,049<br>(69/411)	10,217<br>(12/469)	2,393<br>(10/209)	15,276<br>(58/424)	9,296<br>(6/493)	2,478<br>(8/156)	822<br>(36/68)	33<br>(1/2)	70<br>(3/7)	98,240<br>(293/3504)

[a]In total, 112,593 samples were collected. We isolated 138 viruses from 2,656 goose samples, 22 viruses from 1,347 pigeon samples, but no viruses from the 10,350 wild bird feces. Because H7N9 virus was not isolated from the latter samples, we did not include information about these samples in the table.
