# Supplementary material for: H7N9 virulent mutants detected in chickens in China pose an increased threat to humans
Source: Cell Res. 2017 Oct 24;27(12):1409–21. doi: 10.1038/cr.2017.129 (PMC5717404; doi:10.1038/cr.2017.129)
Supplement: Supplementary information, Table S2 — Amino acids at positions 627 and 701 of the PB2 gene of viruses recovered from the organs of ferrets that were inoculated with the CK/SD008 virusa. [file cr2017129x8.pdf]

Table S2. Amino acids at positions 627 and 701 of the PB2 gene of viruses recovered from the organs of ferrets that were inoculated with the CK/SD008 virus[a].

Animal	Key amino acids in PB2	Viral samples recovered from different organs of ferrets								
		Tonsil	Nasal turbinate	Left lung			Right lung			Brain
				Cranial	Middle	Caudal	Cranial	Middle	Caudal	
Ferret 1	627K	0/10	2/10	0/10	8/10	1/10	1/10	0/10	4/10	0/10
	701N	5/10	0/10	0/10	1/10	0/10	0/10	8/10	0/10	0/10
Ferret 2	627K	1/10	3/10	2/10	0/10	3/10	1/10	0/10	0/10	0/10
	701N	0/10	1/10	0/10	4/10	0/10	0/10	3/10	0/10	0/10

[a]Viral RNAs were extracted from the organs of virus-inoculated ferrets. A 500-nucleotide PB2 fragment covering codons 627 to 701 was amplified and cloned into T vectors. Ten molecular clones from each sample were randomly selected and sequenced. The number on the left of the slash shows the number of clones bearing the indicated amino acid at position of PB2, and the number on the right of the slash shows the total number of clones sequenced.
