# Supplementary material for: H7N9 virulent mutants detected in chickens in China pose an increased threat to humans
Source: Cell Res. 2017 Oct 24;27(12):1409–21. doi: 10.1038/cr.2017.129 (PMC5717404; doi:10.1038/cr.2017.129)
Supplement: Supplementary information, Table S3 — Amino acids at positions 627 and 701 of the PB2 gene of viruses recovered from the nasal wash of ferrets that were inoculated with or exposed toCK/S1053 and CK/SD008. [file cr2017129x9.pdf]

Table S3. Amino acids at positions 627 and 701 of the PB2 gene of viruses recovered from the nasal wash of ferrets that were inoculated with or exposed to CK/S1053 and CK/SD008.

Virus	Ferret	Key amino acids in PB2	Viruses recovered from animals on different days post inoculation (p.i.) or post exposure (p.e.) [a]				
			Day 2 p.i	Day 4 p.i	Day 6 p.i.	Day 8 p.i.	Day 9 p.e.
CK/S1053[b]	Inoculated ferret #3	627K	0/10	0/10	0/10	NA[c]	NA
		701N	0/10	0/10	2/10	NA	NA
CK/SD008	Inoculated ferret #1	627K	1/10	5/10	4/10	7/10	NA
		701N	0/10	0/10	1/10	1/10	NA
	Inoculated ferret #2	627K	1/10	1/10	6/10	/[d]	NA
		701N	0/10	0/10	3/10	/	NA
	Inoculated ferret #3	627K	0/10	3/10	7/10	8/10	NA
		701N	0/10	0/10	2/10	2/10	NA
	Exposed ferret #1	627K	NA	NA	NA	NA	10/10
		701N	NA	NA	NA	NA	0/10

[a]Viral RNAs were extracted from the nasal washes collected from virus-inoculated and virus-exposed ferrets. A 500-nucleotide PB2 fragment covering codons 627 to 701 was amplified and cloned into T vectors. Ten molecular clones from each sample were randomly selected and sequenced. The number on the left of the slash shows the number of clones bearing the indicated amino acid at position of PB2, and the number on the right of the slash shows the total number of clones sequenced.

[b]The indicated PB2 mutations were not detected in the samples recovered from the other five ferrets that were inoculated with the CK/S1053 virus.

[c]NA, virus was not recovered from samples collected on that day.

[d]/, samples were not available to test.
