# Supplementary material for: H7N9 virulent mutants detected in chickens in China pose an increased threat to humans
Source: Cell Res. 2017 Oct 24;27(12):1409–21. doi: 10.1038/cr.2017.129 (PMC5717404; doi:10.1038/cr.2017.129)
Supplement: Supplementary information, Table S4 — Samples collected from Guangdong province in February 2017 and tested for H7N9 influenza viruses. [file cr2017129x10.pdf]

Table S4. Samples collected from Guangdong province in February 2017 and tested for H7N9 influenza viruses.

Sampling site (numbers)	Species	Total samples collected	H7N9 viruses isolated	No. of viruses with insertions in HA/Total[a]
Poultry market (30)	Chicken	855	20	10/21
	Duck	113	0	/
	Goose	30	0	/
	Environment	152	7	5/7
Farm (60)	Chicken	1800	1	0/1
Total	/	2950	28	15/28

[a]The results were obtained from the partial sequence of the HA gene of the viruses.
