# Supplementary material for: H7N9 virulent mutants detected in chickens in China pose an increased threat to humans
Source: Cell Res. 2017 Oct 24;27(12):1409–21. doi: 10.1038/cr.2017.129 (PMC5717404; doi:10.1038/cr.2017.129)
Supplement: Supplementary information, Table S5 — The amino acids in the cleavage site of HA and key amino acids in HA and PB2 related to receptor binding and virulence of H7N9 viruses isolated in 2017. [file cr2017129x11.pdf]

Table S5. The amino acids in the cleavage site of HA and key amino acids in HA and PB2 related to receptor binding and virulence of H7N9 viruses isolated in 2017.

Viruses [a]	Key amino acids in the HA and PB2 genes					IVPI
	HA			PB2		
	Motif at the HA cleavage site	G186V	Q226L	E627K	D701N	
A/chicken/Shanghai/S1053/2013	PKG - - - - R/G	V	L	E	D	0
A/chicken/Guangdong/SD008/2017 (index strain)	PKGKRTAR/G	V	L	E	D	3.0
A/chicken/Guangdong/SD010/2017	PKGKRTAR/G	V	Q	E	D	3.0
A/chicken/Guangdong/SD027/2017	PKGKRTAR/G	V	L	E	D	/
A/chicken/Guangdong/SD028/2017	PKGKRTAR/G	V	Q	E	D	3.0
A/chicken/Guangdong/SD031/2017	PKGKRTAR/G	V	Q	E	D	/
A/chicken/Guangdong/SD032/2017	PKGKRTAR/G	V	L	E	D	/
A/chicken/Guangdong/SD034/2017	PKGKRTAR/G	V	Q	E	D	/
A/chicken/Guangdong/S11523/2017	PKGKRTAR/G	V	L	E	D	/
A/chicken/Guangdong/S12669/2017	PKGKRTAR/G	V	L	E	D	/
A/chicken/Guangdong/S12681/2017	PKGKRTAR/G	V	Q	E	D	/
A/chicken/Guangdong/S12704/2017	PKGKRTAR/G	V	Q	E	D	/
A/environment/Guangdong/S12706/2017	PKGKRTAR/G	V	Q	E	D	/
A/chicken/Guangdong/S12860/2017	PKGKRTAR/G	V	Q	E	D	/
A/chicken/Guangdong/S10131/2017	PK***R***KRTAR/G	V	Q	E	D	/
A/environment/Guangdong/S11464/2017	PK***R***KRTAR/G	V	Q	E	D	/
A/environment/Guangdong/S11526/2017	PK***R***KRTAR/G	V	Q	E	D	3.0
A/chicken/Guangdong/S12123/2017	PK***R***RRTAR/G	V	Q	E	D	/
A/environment/Guangdong/S12141/2017	PK***R***RRTAR/G	V	Q	E	D	/
A/chicken/Guangdong/S12178/2017	PK***R***RRTAR/G	V	Q	E	D	3.0
A/chicken/Guangdong/S12222/2017	PK***R***RRTAR/G	V	Q	E	D	/
A/chicken/Guangdong/S12367/2017	PKGKRIAR/G	V	Q	E	D	3.0
A/environment/Guangdong/S12412/2017	PKGKRIAR/G	V	Q	E	D	/
**A/Taiwan/1/2017[b]**	PK***R***KRTAR/G	V	Q	K	D	/
**A/Qingyuan(Guangdong)/GIRD1/2017[b]**	PK***R***KRTAR/G	V	Q	K	D	/
**A/Guangdong/17SF006/2017 [b]**	PK***R***KRTAR/G	V	Q	K	D	/

[a]The four different colors indicate the four different motifs in the HA cleavage site detected in the H7N9 viruses; Viruses fully sequenced are underlined and included in Fig. 1 and Extended Fig. 1. Human isolates are shown in bold letters. /, not done.

[b]Sequences were obtained from Global Initiative on Sharing Avian Influenza Data (GISAID).
